# Bacteriocins: potentials and prospects in health and agrifood systems

**DOI:** 10.1007/s00203-024-03948-y

**Published:** 2024-04-25

**Authors:** Rine Christopher Reuben, Carmen Torres

**Affiliations:** Area of Biochemistry and Molecular Biology, OneHealth-UR Research Group, University of La Rioja, 26006 Logroño, Spain

**Keywords:** Bacteriocin, Antimicrobial agents, Microbiome, Health, Food, Antimicrobial resistance

## Abstract

Bacteriocins are highly diverse, abundant, and heterogeneous antimicrobial peptides that are ribosomally synthesized by bacteria and archaea. Since their discovery about a century ago, there has been a growing interest in bacteriocin research and applications. This is mainly due to their high antimicrobial properties, narrow or broad spectrum of activity, specificity, low cytotoxicity, and stability. Though initially used to improve food quality and safety, bacteriocins are now globally exploited for innovative applications in human, animal, and food systems as sustainable alternatives to antibiotics. Bacteriocins have the potential to beneficially modulate microbiota, providing viable microbiome-based solutions for the treatment, management, and non-invasive bio-diagnosis of infectious and non-infectious diseases. The use of bacteriocins holds great promise in the modulation of food microbiomes, antimicrobial food packaging, bio-sanitizers and antibiofilm, pre/post-harvest biocontrol, functional food, growth promotion, and sustainable aquaculture. This can undoubtedly improve food security, safety, and quality globally. This review highlights the current trends in bacteriocin research, especially the increasing research outputs and funding, which we believe may proportionate the soaring global interest in bacteriocins. The use of cutting-edge technologies, such as bioengineering, can further enhance the exploitation of bacteriocins for innovative applications in human, animal, and food systems.

**Supplementary Information:**

The online version contains supplementary material available at 10.1007/s00203-024-03948-y.

## Introduction

Antimicrobial resistance (AMR) has risen as one of the major public health challenges in recent times. While the discovery of antibiotics revolutionized modern medicine making them the most successful therapeutic agents to be widely used against bacterial infections, the overuse and misuse of antibiotics have resulted in the emergence of antibiotic-resistant bacteria (Alonso et al. 2017; Torres et al. 2018; Baquero et al. 2021). Regardless of the appropriateness of antibiotics use, routine use of antibiotics at the individual and/or community level exerts immense selective pressure which drives bacterial evolution and the development and acquisition of resistant determinants (Bloom et al. 2018; Baquero et al. 2021). Importantly, antibiotic use in human and veterinary medicine and food production is increasing, and this may likely continue into the coming years as unrestrained antibiotic access rises in resource-limited countries (CDC 2018; Hussain et al. 2020; Gupta 2022). The surge and continuous spread of antibiotic-resistant bacteria and the diminished potency of commercially available antimicrobials and therapeutics necessitate a concerted approach to the search for novel and potent antimicrobials that may become alternatives to available antibiotics. Unless the enigma of widespread AMR and associated public health concerns are urgently prioritized and mitigated, global health and economic burdens will continue to worsen.

Over the past decades, there has been growing interest and research exploring several emerging antimicrobial compounds, including antimicrobial peptides, nanomedicines, probiotics, postbiotics, phytochemicals, bacteriophages, etc. as alternatives to antibiotics (Reuben et al. 2019, 2020; Wang et al. 2020a; Mba and Nweze 2022; Anyaegbunam et al. 2022; Field et al. 2023; Ye et al. 2023; Baquero et al. 2024). Interestingly, ribosomally synthesized peptides of bacterial origin have received increased attention and hold great potential as valuable antimicrobial compounds against a broad spectrum of multi-drug resistant (MDR) pathogens as well as therapeutic agents for the treatment of several diseases (Lynch et al. 2019; Magana et al. 2020; Mba and Nweze 2022; Telhig et al. 2022; García-Vela et al. 2023). Bacteriocins, which are antimicrobial peptides synthesized by bacteria have attracted increasing interest due to their high antimicrobial activities, stability, and low toxicity (Cotter et al. 2013; Lynch et al. 2019; Deslouches et al. 2020; Wiman et al. 2023). The majority of bacteria synthesize at least one known or unknown bacteriocin (Riley and Wertz 2002; Meade et al. 2020; Darbandi et al. 2022). While the ecological function of bacteriocins is yet to be fully elucidated, they are believed to help bacteria outcompete other members of the community, modulate the competitive landscape through direct or exclusive antagonisms, and also serve as signaling molecules (Dobson et al. 2012; Meade et al. 2020; Darbandi et al. 2022).

Bacteriocins are structurally diverse and encoded by highly variable and complex biosynthetic gene clusters that evolve rapidly (Cotter et al. 2013; Heilbronner et al. 2021; Ye et al. 2023). The high antimicrobial activity, diversity, low toxicity, stability, and therapeutic benefits of bacteriocins have prompted a soaring multi-sectorial and transdisciplinary interest in their search, characterization, and broad applications either as (i) antimicrobial/therapeutic compounds for the treatment and prevention of human and animal diseases, (ii) food additives for the inhibition of foodborne pathogens or spoilage organisms, (iii) feed supplements for growth promotion in animal production, or (iv) microbiome-based interventions for the modulation of the gut, reproductive tract, respiratory tract, skin, and food microbiomes (Cotter et al. 2013; Jayaraman et al. 2013; Campion et al. 2013; Vieco-Saiz et al. 2019; Sarika et al. 2019; Liu et al. 2020; Soltani et al. 2021a; Bosák et al. 2021; Saur et al. 2021; Polak et al. 2021). Bacteriocins have a high potential for medico- and techno-economic use in biomedicine and agri-food systems, thus depicting their relevance and prospects across the One Health continuum.

From a cross-disciplinary and multisectoral perspective, several bacteriocins from both Gram-negative and Gram-positive bacteria are being explored. Some of these bacteriocins have shown great potential and prospects for field applications. Bacteriocin research has evolved from basic characterization to high-throughput identification and applications in multiple systems. The increasingly comprehensive scientific reports of multi-sourced (and novel) bacteriocins as well as their impacts on human and animal health, food quality and safety, [micro]ecological landscapes, and industry necessitate their unified compilation and synthesis. Furthermore, bacteriocin research and bibliographies are often disjointed in a ‘stand-alone’ manner seldom without a nexus linking them across disciplines. Understanding the current bacteriocin research across disciplines will inform concerted future research direction which may further foster interdisciplinary perspectives and collaborations. To this end, this review carefully assessed and compiled significant advances and emerging roles of bacteriocins and related innovations within the One Health continuum. Furthermore, we provided a comprehensive cross-disciplinary, multisectoral, and up-to-date potential and prospects of bacteriocins applications and bibliometrics in the human, animal, and food systems.

## Advances in the biology, classification, and sources of bacteriocins

Bacteriocins have been generally defined as ribosomally synthesized antimicrobial peptide molecules that can either be enzymatically modified or remain unaltered (Cotter et al. 2013; Johnson et al. 2018; Simons et al. 2020; Heilbronner et al. 2021). They are abundant and highly diverse with widespread synthesis among different groups of bacteria (Riley and Wertz 2002; Cotter et al. 2013; Fernández-Fernández et al. 2023c). It has been suggested that 30% to 99% of Archaea and bacterial species synthesize one or more bacteriocins (Klaenhammer 1988; Riley 1998). Typically, bacteriocins have a narrow spectrum of bactericidal or bacteriostatic activity against taxonomically related bacteria (O’Connor et al. 2018; Simons et al. 2020; Darbandi et al. 2022), but occasionally they can have a broad spectrum of activity against unrelated bacteria (Cotter et al. 2005; Mills et al. 2011; Silva et al. 2018). The biosynthetic mechanisms for these antimicrobial peptides are relatively simple and often encoded in transferable elements such as plasmids and transposons (Klaenhammer 1993; And and Hoover 2003; Fernández-Fernández et al. 2023b). Bacteriocins are synthesized as biologically inactive precursor peptides harboring an N-terminal leader sequence (Kanmani et al. 2013; Liu et al. 2023). These precursor peptides are often detached from the leader peptide and exported outside the cell after post-translational modifications (PTMs) (Riley and Wertz 2002; Mokoena 2017; Soltani et al. 2021a). Bacteriocinogenic bacteria have developed mechanisms to protect themselves from being killed by the bacteriocins they produce. These mechanisms include using efflux pumps to export bacteriocins from inside the cells to the outside, synthesizing self-immunity proteins, or using both mechanisms in some instances (Bastos et al. 2015; Ben Lagha et al. 2017; Bountra et al. 2017).

The function of bacteriocins depends on the recognition of specific receptors and ionic interactions with the hydrophobic surface molecules of target cells (Soliman et al. 2010; Todorov et al. 2022; Śmiałek-Bartyzel et al. 2023). This is typically considered the initial step of the antimicrobial mechanism of action exerted by bacteriocins. To infiltrate the cell membrane and compromise cellular integrity, bacteriocins must effectively recognize these receptors and also express physicochemical interactions with the target cells. For example, receptors like mannose phosphotransferase and lipid II are primarily recognized by class II, unmodified bacteriocins (such as pediocin PA-1 and enterocin CRL35) and class I, post-translationally modified bacteriocins (RiPPs) (such as nisin and mutacin 1140), respectively (Grein et al. 2019; Wang et al. 2020c; Zhu et al. 2022). These intricate interactions between bacteriocins and target cells are often influenced by various physicochemical factors such as temperature, pH, and other chemical constituents. These factors also affect cell membrane integrity and physiological conditions, which consequently impact bacteriocin interactions with specific receptors or directly with the cell membrane (Todorov et al. 2022). Depending on their primary structure and complexity, bacteriocins exert antimicrobial activity through distinct mechanisms of action on susceptible microbial strains. Some bacteriocins cause cell lysis by inhibiting cell wall synthesis or forming pores in the cell membrane. Others act inside the target cells, inhibiting protein production and gene expression (Dobson et al. 2012; Darbandi et al. 2022).

Since the discovery of bacteriocins about a century ago, there has been an increasing number of characterized and identified bacteriocins. These bacteriocins are heterogeneous and highly diverse, possessing a wide range of complexities, structures, sizes, mechanisms of action, spectra of activity, and target cells. To better collate and understand the structural and functional diversities of bacteriocins, some integrated open-access databases and tools have been developed. These include antiSMASH 2.0 [http://antismash.secondarymetabolites.org/ (Blin et al. 2013)], BAGEL3 [http://bagel.molgenrug.nl/ (van Heel et al. 2013)], ADAM, [http://bioinformatics.cs.ntou.edu.tw/ADAM (Lee et al. 2015)], BACTIBASE, [http://bactibase.hammamilab.org (Soltani et al. 2021a)], NucleBact [https://pubmlst.org/projects/nuclebact (Sharp et al. 2017)], LABiocin [https://bio.tools/LABiocin_database (Kassaa et al. 2019)], BUR—bacteriocins database URMITE [https://drissifatima.wixsite.com/bacteriocins (Drissi et al. 2015)], Bacteriocin (https://aapep.bocsci.com/), and Syngulon (https://syngulon.com/). Following the first bacteriocin classification by Klaenhammer (1993), several classifications have been proposed and used in recent years. Due to the advent of cutting-edge high throughput technologies and new developments in bacteriocins’ structures, functions, and mechanisms of action, the classification of bacteriocins progressively evolved, undergoing continuous modification. These classification systems primarily hinge on multiple factors such as physical properties, chemical structure, molecular composition, size, stability, mechanism of action, post-translational modification, microbial target, organism producing them, and cell wall type (Klaenhammer 1993; Dobson et al. 2012; Arnison et al. 2013; Cotter et al. 2013; Bastos et al. 2015; Alvarez-Sieiro et al. 2016; Johnson et al. 2018; Soltani et al. 2021a).

**Fig. 1:**
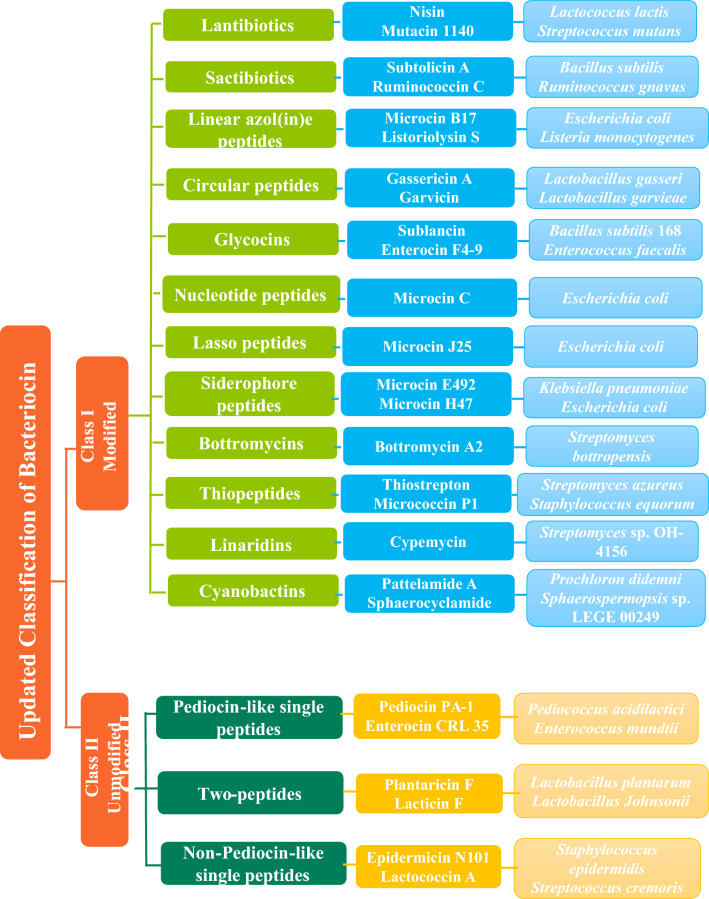
Updated classification of bacteriocins based on post-translationally modified and unmodified peptides (Adopted from Mills et al. 2017; Soltani et al. 2021a)

Building on the previous classification (Cotter et al. 2013) and recent advances in ribosomally synthesized and post-translationally modified peptides (RiPPs), the latest and updated classification system proposed by Soltani et al. (2021a) suggests two large classes of bacteriocins. Class I, also referred to as RiPPs have molecular masses < 5 kDa and contain post-translational modifications (PTMs). Class I is subdivided into 12 subclasses, including lanthipeptides, sactipeptides, linear azole(ine)-containing peptides (LAP), circular peptides, glycocins, nucleotide peptides, lasso peptides, siderophore peptides, and Bottromycins from both Gram-positive and Gram-negative bacteria (Cotter et al. 2013; Norris and Patchett 2016; Mills et al. 2017). Additionally, thiopeptides and linaridins from Actinobacteria (Bagley et al. 2005; Claesen and Bibb 2010), and cyanobactins produced by different cyanobacteria (Martins and Vasconcelos 2015; Martins et al. 2018) are subclasses of class I bacteriocin. Class II bacteriocins, also known as unmodified bacteriocins, have molecular masses < 10 kDa and are subdivided into three subclasses: pediocin-like bacteriocins (single peptides containing the YGNGV consensus sequence), two peptides bacteriocins (containing two or more unmodified peptides), and non-pediocin-like bacteriocins (unmodified linear single peptides devoid of the YGNGV) (Mills et al. 2017; Soltani et al. 2021a) (Fig. 1). Generally, the PTMs make class I bacteriocins more stable to extreme pHs, high temperatures, or proteolysis than class II bacteriocins. However, the presence of disulfide bridges in class II bacteriocins relatively increases their stability (Soltani et al. 2021a).

Bacteriocins are abundant and heterogeneous in nature. Bacteriocin-producing bacteria can be found in both conventional and unconventional sources. While the human gut is considered a conventional source of bacteriocinogenic bacteria, unconventional sources include soil, water, foods/food products, animal guts, and the vagina and nose of animals and humans (Ryan et al. 2008; Vera Pingitore et al. 2009; Lo Verso et al. 2018; Zielińska and Kolożyn-Krajewska 2018; Fuochi et al. 2019; Reuben et al. 2020; Darbandi et al. 2022; Fernández-Fernández et al. 2023a, b, c, d; Navarro et al. 2023). Common bacteriocin-producing bacteria in humans include *Enterococcus*, *Escherichia coli*, *Lactobacillus*, *Lactococcus*, *Pediococcus*, *Staphylococcus*, and *Streptococcus* (Ryan et al. 2008; Lakshminarayanan et al. 2013; Zalewska et al. 2018; Laux et al. 2019; Kassem et al. 2021; Darbandi et al. 2022). These bacteria not only act as the first line of defense against invading pathogens, but their bacteriocins also play a role in enhancing the immune system (Zipperer et al. 2016; O’Sullivan et al. 2019).

Interestingly, most of the bacteriocins that have been successfully characterized and identified are produced by lactic acid bacteria (LAB), which are frequently found in milk and dairy products. LAB is a diverse group of bacteria that has garnered significant interest due to their widely recognized safety status, known as ‘Generally Recognized as Safe’ (GRAS) and ‘Qualified Presumption of Safety’ (QPS) status (Reuben et al. 2020; Zimina et al. 2020). Some well-known bacteriocinogenic bacteria commonly found in dairy products include *Lactococcus lactis* and *Lactobacillus plantarum* (found in camel, cow, and goat milk), *Lactobacillus kefiranofaciens* and *L. plantarum* (found in cheese and kefir), and *Lactobacillus brevis*, *Enterococcus* spp., and *Streptococcus thermophilus* (found in other dairy products) (Reuben et al. 2020; Zimina et al. 2020; Benkirane et al. 2022). *Lactobacillus acidophilus* is commonly isolated from yogurt and fermented soy products as a bacteriocin-producing bacterium, while *Bifidobacterium lacti*s and *Brevibacillus brevis* are most commonly found in raw milk (Darbandi et al. 2022). In milk products, *Lactobacillus*, *Lactococcus*, and *Streptococcus* are the predominant bacteriocin-producing bacteria.

From fermented raw or cooked meat products, *Lactobacillus brevis*, *Lactobacillus curvatus*, *Lactobacillus fermentum*, *Lactobacillus plantarum* subsp. *plantarum*, *Enterococcus faecium* UAM1, *Pediococcus pentosaceus*, and *P. accidilactici* are widely isolated bacteriocinogenic bacteria (Aymerich et al. 2011; Zielińska and Kolożyn-Krajewska 2018; Khorshidian et al. 2021; García-López et al. 2023; Kaveh et al. 2023). These bacteria exhibit inhibitory activity against major foodborne pathogens including *Aeromonas hydrophila*, *Listeria monocytogenes*, and *Staphylococcus aureus*, thereby preventing their growth in meat products (Winkowski and Montville 1992; Khan et al. 2016). *E*. *faecium* HL7, *L*. *plantarum*, and *L*. *brevis* LAP2 are commonly associated with fish and seafood (Vijayabaskar and Somasundaram 2008; Gómez-Sala et al. 2015; Ringø et al. 2018), while *L*. *brevis*, *L. paracasei*, *L. pentosus*, *L. fermentum*, *L. plantarum*, *Weissella*, *Pediococcus*, and *Enterococcus durans* are known bacteriocin-producing bacteria found in fruits and vegetables (Knorr 1998; Linares-Morales et al. 2020). Soil is another extensively studied unconventional source of bacteriocinogenic bacteria. Many bacteriocins obtained from soilborne bacteria and rhizosphere exhibit inhibitory and biocidal activity against phytopathogens, pests, and insects, making them useful for plant protection as well as biopesticides, bioinsecticides, and growth stimulants (Lv et al. 2017; Zimina et al. 2020). Soil bacteria, including *Pseudomonas putida* BW11M1, *Bacillus subtilis* 14B, and *Clavibacter michiganensis* subsp. michiganensis (*Cmm*) produce bacteriocin putidacin, Bac 14B, and michiganin A which have inhibitory activity against *P. putida* GR12-2R3, *Agrobacter tumefaciens*, and *C. michiganensis* subsp. *Sepedonicus*, the etiological agents of plant diseases. Similarly, *Bacillus clausii* GM17 produces bacteriocin Bac GM17 which has broad-spectrum antifungal and antibacterial activity against multiple phytopathogens (Zimina et al. 2020). Recently, our group characterized and identified different bacteriocins of staphylococcal origin from multiple sources including humans, food, migratory birds, pets, wild animals, and the environment (Fernández-Fernández et al. 2022a, b, 2023a; b).

## Trends in bacteriocins research: a bibliometrics perspective

**Fig. 2:**
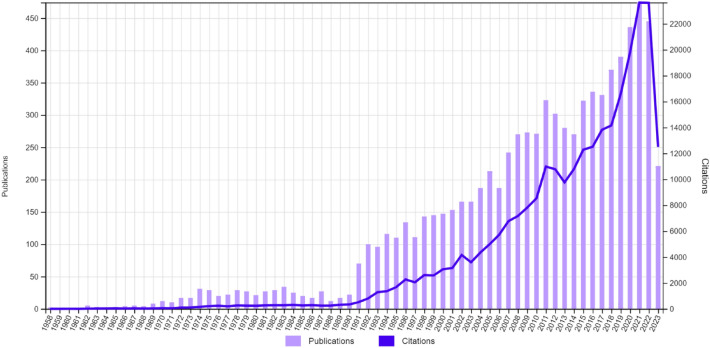
Bibliometric indices of bacteriocin-related publications and citations

To fully comprehend the current direction of bacteriocin research, we conducted a bibliometric analysis to identify the prevalent research trends and gaps in the field as well as future research perspectives. In August 2023, we conducted a comprehensive literature search on the Web of Science core collection database (http://www.webofscience.com/) using the keyword ‘bacteriocin’ to identify relevant bacteriocin-based publications. We included articles published in 16 different languages until August 2023 for our synthesis (Table S1). In total, there were 8303 publications with 270,493 citations recorded in the Web of Science (WoS) core collection between 1958 to August 2023. Throughout this period, we observed a relatively steady increase in the number of articles and citations, with a notable spike in 2021 (articles = 474; citations = 23,638) (Fig. 2). It is worth mentioning that the last decade has seen an unprecedented exponential increase in bacteriocin-related research, nearly doubling the total research output of previous decades. Given the utilization of advanced technologies in bacteriocin research and the growing global interest and acceptance of bacteriocins in recent years, this trend is not surprising.

There is a wide geographical spread of bacteriocin-related research outputs, spanning 127 countries or regions around the world. The United States, China, and Spain are leading with 1238 (14.907%), 716 (8.621%), and 605 (7.285%) publications respectively, while 26 other countries have over 100 publications each (Table 1). Interestingly, the top regions leading in bacteriocin-related research outputs are North America, Europe, and Asia which are known to have highly industrialized economies. The dominance of these countries can be rationalized by the public perception and national/regional approval of certain bacteriocins for commercial use. For example, the European Union (EU) approved the use of Nisin (E 234) as a food additive in various food categories in the EU under Directive 83/463/EEC, Directive 95/2/EC in 1988, and EU Annex II of Regulation (EC) 1333/2008 in 2006, following its safety evaluation by the European Food Safety Authority expert panel (European Food Safety Authority (EFSA) 2006). Similarly, the Food and Drug Administration of the USA approved the use of nisin as an antimicrobial agent in 1988 (and later amended at 59 FR 14364, Mar. 28, 1994; 68 FR 24879, May 9, 2003; and 88 FR 17724, Mar. 24, 2023) (https://www.accessdata.fda.gov/scripts/cdrh/cfdocs/cfcfr/cfrsearch.cfm?fr=184.1538), and it was given the GRAS status for use in processed food (Cotter et al. 2005; Shin et al. 2016). The periods of these approvals coincided with the rise in antimicrobial resistance to commercially available antimicrobials and concerns about the use of in-fed antimicrobials in livestock production (European Commission 2005).

**Table 1:** Country-specific bacteriocins-related research outputs (top 50)

Countries/Regions	Record count	% of 8303
USA	1238	14.91
China	715	8.611
Spain	605	7.287
India	522	6.287
France	500	6.022
Brazil	478	5.757
Japan	431	5.191
Canada	418	5.034
Germany	331	3.987
South korea	318	3.83
Ireland	281	3.384
Norway	280	3.372
Italy	278	3.348
England	236	2.842
The Netherlands	204	2.457
Argentina	196	2.361
Belgium	191	2.3
Turkey	163	1.963
Iran	144	1.734
Egypt	135	1.626
South africa	135	1.626
Thailand	135	1.626
Slovakia	131	1.578
Australia	126	1.518
New Zealand	126	1.518
Denmark	108	1.301
Pakistan	108	1.301
Russia	106	1.277
Malaysia	105	1.265
Poland	98	1.18
Portugal	98	1.18
Tunisia	92	1.108
Mexico	90	1.084
Greece	86	1.036
Czech Republic	83	1.000
Switzerland	80	0.964
Taiwan	64	0.771
Indonesia	59	0.711
Finland	57	0.686
Bulgaria	53	0.638
Saudi Arabia	52	0.626
Scotland	48	0.578
Serbia	47	0.566
Nigeria	46	0.554
Slovenia	46	0.554
Algeria	43	0.518
Sweden	43	0.518
Chile	40	0.482
Israel	34	0.409
Morocco	34	0.409

**Fig. 3:**
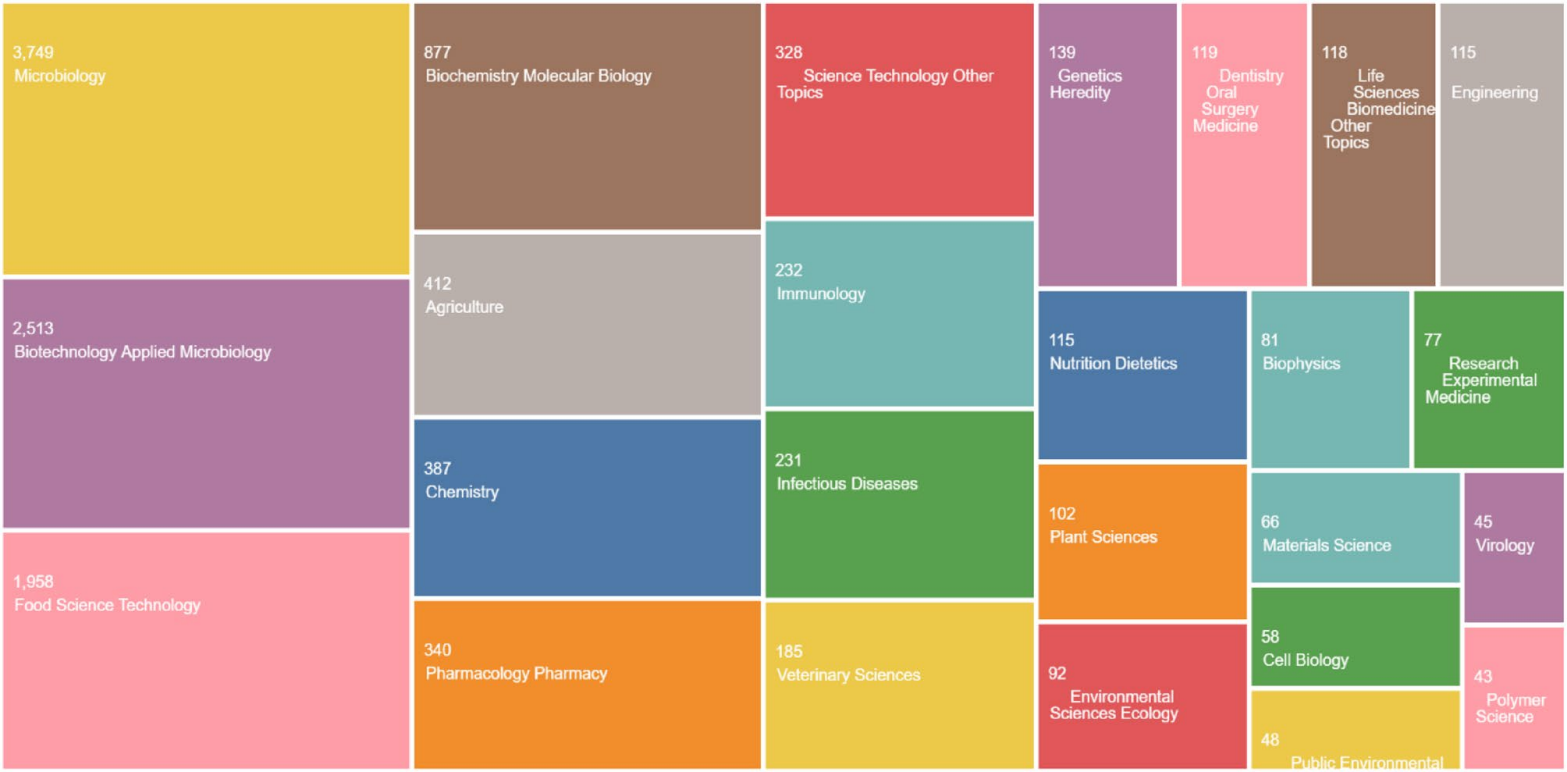
TreeMap representation of bacteriocin-related publications across disciplines

The categorization of publications related to bacteriocins, according to disciplines and specialties demonstrates the broad and multidisciplinary nature of bacteriocin research and applications in various fields, including the One Health systems. Out of the 8303 publications, 45.1% (3749), 30.3% (2513), and 23.6% (1958) were categorized under microbiology, biotechnology and applied microbiology, and food science and technology, respectively. Other disciplines that have significant bacteriocin-related research outputs, with over 100 publications, include pharmacology and pharmacy, biochemistry and molecular biology, agriculture, infectious diseases, immunology, nutrition and dietetics, plant sciences, chemistry, veterinary sciences, dentistry and oral medicine, and multidisciplinary sciences (Fig. 3). Microbiology is the discipline with the highest number of research outputs, which is expected since bacteriocins are microbial products. Therefore, most microbiological research focuses on characterizing, synthesizing, and identifying (novel) bacteriocins from various microorganisms isolated from both conventional and unconventional sources. Biotechnology and applied microbiology, as well as food science and technology, are also prominent research areas in bacteriocins-related publications, highlighting the dynamic and diverse biotechnological applications of bacteriocins and their increasing use in food production (Gharsallaoui et al. 2016; Chandrakasan et al. 2019). Furthermore, emerging areas with bacteriocin-related publications include obstetrics and gynecology, dermatology, oncology, soil science, nanoscience and nanotechnology, neurosciences, entomology, and agronomy. To further support the categorization of bacteriocin-associated research outputs, we examined the intra-discipline citations at both the meso- and micro-scale. Our findings revealed that inflammatory bowel diseases and infections (3325), bacteriology (787), antibiotics and antimicrobials (345), dentistry and oral medicine (236), and plant pathology (187) were the specialized areas with the highest number of citations (Figure S1).

While Elsevier (1723), Springer Nature (1202), Wiley (914), the American Society for Microbiology (905), and MDPI (323) are the publishers with the most bacteriocin-related publications, the United States Department of Health and Human Services (HHS), the National Institutes of Health* (*NIH), National Natural Science Foundation of China (NSFC), the Brazilian National Council for Scientific and Technological Development (CNPQ), and the Spanish Government are among the leading funding agencies for bacteriocin research (Tables S2 and S3). There is a global spread of funding for bacteriocin research. This demonstrates the willingness of funding agencies across different regions of the world to support bacteriocin research. Finally, among the 17 Sustainable Development Goals (SDGs), 7277, 172, 123, 63, and 46 bacteriocin-related research outputs primarily align with Goal 3: Good Health and Well-being, Goal 2: Zero Hunger, Goal 15: Life on Land, Goal 13: Climate Action, and Goal 12: Responsible Consumption and Production (Table S4). While there are a few bacteriocin research outputs that align with several other SDGs, this highlights the potential of bacteriocins in promoting global peace and prosperity for both people and the planet, both now and in the future (https://sdgs.un.org/goals).

## Bacteriocins vs. viable [probiotics/protective cultures] cells: mitigating emerging concerns

Most bacteriocin-producing bacteria, especially (foodborne) LAB and gut commensals are widely used as probiotics or protective cultures in food production and as supplements for animals and humans. Bacteriocin production has long been recognized as an important trait in probiotics or protective cultures (Corr et al. 2007; Dobson et al. 2012; Cotter et al. 2013). Although the exact ecological function of bacteriocins is not fully understood, it is believed that they play a significant role in the functionality of probiotics within their host. Functioning as colonizing peptides, bacteriocins facilitate the colonization and dominance of a producing [probiotic] strain into an already established niche (Riley and Wertz 2002; Anjana 2022). These promising advantages exerted by bacteriocins are attributed to their biofunctional properties and structural diversity (Zhu et al. 2023; Wang et al. 2023). Current research focuses on exploring the underlying bioactivity of bacteriocins in the development of novel probiotics for broad and newer applications in biomedicine and the agri-food industry. Under different conditions, probiotic-derived bacteriocins are often evaluated and used alone or in combination with the producing strains (Umair et al. 2022; Hussien et al. 2022; Ahn et al. 2023; Mihailovskaya et al. 2023; Yu et al. 2023). Bacteriocins can directly inhibit pathogens and other competing microorganisms (Majeed et al. 2011; Simons et al. 2020) or modulate the composition and diversity of microbial communities and the host immune system through signaling mechanisms (Czárán et al. 2002; Di Cagno et al. 2007; Chikindas et al. 2018). For example, vancomycin-resistant enterococci (VRE) were successfully controlled using pediocin PA-1-producing *P. acidilactici* MM33. Conversely, no effect was recorded using the non-pediocin PA-1 producing *P. acidilactici* MM33 strain (Millette et al. 2008). In separate studies, novel bacteriocins such as cerein B4080, cerein 7B, bacteriocin AS-48, garvicin KS, and micrococcin P1 were studied and proposed as promising alternatives for the treatment of skin and soft tissue infections caused by multidrug-resistant *Staphylococcus aureus* (Ovchinnikov et al. 2020; Velázquez-Suárez et al. 2021; Jaumaux et al. 2023). Like probiotics, the antimicrobial properties of bacteriocins are pathogen-specific and activity-dependent (Tran et al. 2023; Zhu et al. 2023). Similarly, oral administration of bacteriocin (ABP118) producing *L. salivarius* UCC118 reportedly controlled *L. monocytogenes* infection than the non-ABP118 producing strain of *L. salivarius* UCC118 (Corr et al. 2007). Therefore, assessing the antimicrobial profiles of bacteriocins against multiple pathogens under different conditions is a prerequisite for their selection in the treatment of antibiotic-resistant pathogens in clinical settings.

The common and primary denominator in probiotics and protective cultures is the viability of the cells. Both probiotics and protective cultures essentially consist of live or viable cells specifically selected to confer desired benefits when used in adequate amounts (Hill et al. 2014; Hammami et al. 2019; Fischer and Titgemeyer 2023). However, emerging evidence demonstrates their viability as a non-essential precursor for exerting the desired beneficial properties. Some non-viable components from probiotics and protective cultures, such as bacteriocins and postbiotics, can exude comparable beneficial properties (Raman et al. 2016; Hammami et al. 2019; Homayouni Rad et al. 2021; Mack et al. 2022; Liang and Xing 2023; Teng et al. 2023). Bacteriocins are highly diverse and often outperform viable cells in terms of safety, bioavailability, absorption, distribution, and metabolism while maintaining cognate bioactivities (Ng et al. 2020; Todorov et al. 2022; Liang and Xing 2023).

In recent decades, there have been overwhelming concerns associated with the use of probiotics and protective cultures, particularly regarding the acquisition and distribution of undesired genes, such as antibiotic resistance and virulence. In most cases, microbial strains used as probiotics or protective cultures are generally benign and pose no risk. However, untoward conditions, especially horizontal transfer directly or indirectly predispose them to acquire or spread antibiotic resistance and virulence genes among the commensal microbiota (and opportunistic pathogens) inhabiting the same niche (Imperial and Ibana 2016; Costa et al. 2018; Kothari et al. 2019). Human or animal microbiota is believed to be a trove of numerous [functional] genes, including antibiotic resistance genes which can be easily shared or transferred between resident and transient bacteria (e.g., probiotics and pathogens) (Kothari et al. 2019). Several studies have extensively documented the transfer of undesirable genes between the resident microbiota (in the gut or food) and the strains used as probiotics or protective cultures (Hu et al. 2013; Aarts and Margolles 2014; Abriouel et al. 2015; Wolfe 2023; Sada et al. 2024). Other concerns associated with the use of probiotics and protective cultures include deleterious metabolic activities and imbalances, the eruption of excessive immune responses, persistent microbial colonization that disrupts the normal microbiota, septicemia, and localized or systemic infections (Spano et al. 2010; Doron and Snydman 2015; Pararajasingam and Uwagwu 2017; Costa et al. 2018; Kim et al. 2018; Sada et al. 2024).

The concerns associated with the use of live cells necessitate the use of bacteriocins, which may be safer for application in multiple systems. Since most microbial strains used as probiotics or protective cultures produce known (or unknown) bacteriocins or bacteriocin-like inhibitory substances (BLIS) that exert similar beneficial effects as the viable strains, it is believed that researchers may sooner than expected preferably explore the use of bacteriocins to mitigate the concerns associated with the use of viable cells. Bacteriocins seem to pose little or no risks for use within animal, human, and food systems while exerting their heterogeneous beneficial effects. Therefore, they may be the most preferred choice for broad applications earlier than anticipated. To fully understand the risks associated with the use of viable cells and to establish the preference for bacteriocin applications in real-life situations, more comprehensive studies using experimental evolution across multiple systems are required.

## Applications of bacteriocins

As the science of bacteriocins steadily progresses, their areas of application are increasing proportionately, encompassing previously unknown areas. Since their discovery, bacteriocins have been used to improve food production, preservation, and safety in the food industry. However, their potential has now extended to various fields, including biotechnology, ecology, pharmaceuticals, agriculture, clinical settings, and veterinary medicine. Bacteriocins offer sustainable solutions to a wide range of scientific problems. Here, we critically evaluated and compiled the significant advances and emerging roles of bacteriocins as well as the latest bacteriocin-related innovations aimed at harnessing their heterogeneous potential and prospects for multisectoral applications in health and agrifood systems. Table 2 summarizes some bacteriocins with potential applications in different systems.

**Table 2:** Bacteriocins with potential applications

Bacteriocin	Producer	Microbiome modulation		
		Effect	Model	References
Nisin Z	*Lactococcus lactis*	Reduction of enteric pathogens	Mouse	Millette et al. (2008)
*Nisin*	*L. lactis*	Modulation of microbiome-brain-gut axis neurochemicals	Mice	Jia et al. (2018)
*Nisin Z*	*L. lactis*	Reduction of intestinal colonization of vancomycin-resistant enterococci (VRE) and Immunomodulatory effect	Murine	Millette et al. (2008)
Nisin P	*L. lacti*s SMN003	Reduction of *S. aureus* and regulation of cytokine concentration to reduce uterine inflammation in rats	Rat	Dabour et al. (2009)
*Nisin*	*L. lactis*	Control of meningitis, sepsis, and pneumonia	In vitro and mouse	Goldstein (1998)
Nisin A	*L. lactis*	Decrease the levels of IL-6, IL-8, and TNF-α and the growth of bacteria wound	Ex vivo	Mouritzen et al. (2019)
Sakacin A (SakA), pediocin PA-1 (PedPA-1), enterocins P, Q and L50 (enterocins), plantaricins EF and JK (plantaricins) and garvicin ML (GarML)	Multiple bacteriocinogenic strains	Modulation of the abundance of gut microbiota and structure	Mice	Umu et al. (2016)
Bactofencin A	*Lactobacillus salivarius* DPC6502	Modulation of gut microbial populations	Simulated colon	Guinane et al. (2016)
Bactofencin A	*L. salivarius*	Reduction of *Listeria* and staphylococcal counts	In vitro	O’Connor et al. (2018)
*Bactofencin A*	*L. salivariu*s DPC6502	Increase relative abundances of *Bifidobacterium* and *Streptococcus* while lowering the abundances of *Blautia* and *Clostridium* spp.	Mice	Sun et al. (2020)
Lacticin3147	*L. lactis* DPC3147	Reduction of *Clostridium difficile* associated diarrhea (CDAD)	In vitro	Rea et al. (2007)
*Lactocin 160*	*L. rhamnosus*	Control *Escherichia coli* and *Bordetella pertussis*	In vitro	Belfiore et al. (2007)
Bacteriocin Abp118	*L. salivarius*	Reduction of Listeriosis	Murine and pigs	Riboulet-Bisson et al. (2012)
Bacteriocin OR-7	*L. salivarius* NRRLB	Reduction of *Campylobacter jejuni* counts	Chicken	Ilinskaya et al. (2017)
Erwinaocin NA4	*Erwinia carotovora* NA4	Reduction of coliphage	In vitro	Dey et al. (2021)
Pediocin PA1	*Pediococcus acidilactici*	Control listeriosis	Mouse	Dabour et al. (2009)
Pediocin AcH	*P. acidilactici*	Reduction of enteric pathogens	Mouse	Millette et al. (2008)
Enterocin A/P	*Enterococcus faecium* P13	Modulation of gut microbiota, improving growth and immune response	Rabbit	Pogány Simonová et al. (2022)
Microcin M	*Escherichia coli* MC4100	Inhibition of intestinal pathogenic bacteria and reduction of intestinal inflammation	Mice	Sassone-Corsi et al. (2016)
Microcin J25	*E. coli*	Modulation of porcine microbiota composition and metabolome	PolyFermS in vitro continuous fermentation	Naimi et al. (2022)
Microcin J25	*E. coli*	Improve intestinal microbiota and inflammation of broiler and mouse caused by *Salmonella* and Enterotoxigenic* E. coli*	Broiler and mouse	Yu et al. (2018), Wang et al. (2020b)
Gassericin A	*L. gasseri* LA39	Increase relative abundances of beneficial lactic acid bacteria, promote fluid absorption, and decrease diarrhoea	Early weaned piglets	Hu et al. (2018)
Lmo2776	*Listeria monocytogenes*	Target the commensal *Prevotella copri* and modulation of intestinal infection	Mice	Rolhion et al. (2019)
Salivaricin LHM	*L. salivarius*	Antibacterial, immunomodulatory, and antibiofilm	Simulated urinary tract infection	Mahdi et al. (2019)
Plantaricin EF	*L. plantarum*	Intestinal microbial modulation, maintains epithelial barrier integrity, reduction of obesity and fat inflammation	In vitro and mice	Heeney et al. (2019)
Sublancin	*Bacillus subtilis* 800	Protection against methicillin-resistant *Staphylococcus aureus* (MRSA) and enhancement of macrophage function	Mice	Wang et al. (2018, 2019b)

Bacteriocin	Producer	Bacterial infections		
		Target microorganism	Model	References
Bacteriocin C2-1	*Ligilactobacillus salivarius* C2-1	*Listeria monocytogenes* CICC 21633	In vitro	Mu et al. (2024)
Lactocin AL705	*L. curvatus*	*L. monocytogenes*	In vitro	Melian et al. (2019)
Lactocin 160	*L. Rhamnosus*	*Gardnerella vaginalis*, *Bacillus pertussis*	In epivaginal	Turovskiy et al. (2009)
Lacticin NK34	*L. lactis*	*S. aureus/S. simulans*	Mice	Kim et al. (2010)
Thiostrepton	*Streptomyces* spp.	*Mycobacterium abscessus*	In vitro and zebrafish (FDA approved)	Rodnina et al. (1999), Kim et al. (2019)
Thuricin CD	*Bacillus thuringiensis* DPC 6431	*Clostridium difficile*, *L. monocytogenes*	In vitro and mice	Rea et al. (2010, 2014)
Nisin	*L*. *lactis*	*Staphylococcus aureus*, *C. difficile*	In vitro, mice and rat (FDA approved)	Brand et al. (2010), Lay et al. (2016)
Nisin F	*L. lactis* subsp. *lactis*	*S. aureus*	Immunosuppressed Wistar rat	De Kwaadsteniet et al. (2009)
Nisin V	*L. lactis* NZ9700	*L. monocytogenes*	BALB/c mice	Campion et al. (2013)
Mutacin B-Ny266	*S. mutans*	*S. aureus*, *Neisseria*, *Helicobacter*	In vitro and mice	Mota-Meira et al. (2000, 2005)
Mersacidin	*Bacillus* spp. HIL-Y85/54728	Methicillin-resistant *S. aureus* (MRSA)	In vitro and mice	Brötz et al. (1998), Kruszewska et al. (2004)
Mersacidin	*Bacillus* spp. strain HIL Y-85	MRSA	*BALB/cA* mice	Kruszewska et al. (2004)
Plantaricin NC8 αβ (PLNC8 αβ)	*L. plantarum*	*Staphylococcus* spp.,* Porphyromonas gingivalis*	In vitro	Bengtsson et al. (2020)
R-pyocins	*P. aeruginosa*	*Pseudomonas aeruginosa*	In vitro	Redero et al. (2018)
Lassomycin	*Lentzea kentuckyensis*	*Mycobacterium tuberculosis*	In vitro	Gavrish et al. (2014)
Enterocin AS-48	*E. faecalis*	*M. tuberculosis*	In vitro and macrophages	Aguilar-Pérez et al. (2018), Cebrián et al. (2019)
Durancin 61A	*E. durans* 61A	*C. difficile*, vancomycin-resistant enterococci, MRSA, *L. innocua*	In vitro	Hanchi et al. (2016, 2017)
Ruminococcin C	*Ruminococcus gnavus* E1	Pathogenic clostridia and MDR strains	In vitro	Chiumento et al. (2019), Balty et al. (2019)
Gallidermin/epidermin	*S. gallinarum*	*S. epidermidis*, *S. aureus*	In vitro	Bengtsson et al. (2018)
Haemocin type B	*Haemophilus haemolyticus*	*Haemophilus influenza*	In vitro	Latham et al. (2017)
Gassericin E	*L. gasseri* EV1461	Pathogens associated with vaginosis	In vitro	Maldonado-Barragán et al. (2016)
ABP-118	*Lactobacillus salivarius* UCC118	*L. monocytogenes*	Mouse	Corr et al. (2007)
Colicin E1 and Ib	*E. coli* H22	*E. coli* and *Enterobacter* spp.	Mouse	Cursino et al. (2006)
Colicin FY	*E. coli*	*Yersinia enterocolitica*	Mice	Bosák et al. (2012, 2018)
Microcin C7	*E. coli* H22	*Shigella flexneri*	Mouse	Cursino et al. (2006)
Microcin B17	*E. coli Nissle* 1917	*Salmonella* Typhimurium, *S. flexneri*, *E. coli*	Infants and toddlers	Henker et al. (2007)
Micrcoccin P1	*Staphylococcus* spp.	MRSA	In vitro	Fernández-Fernández et al. (2023c)
Unnamed bacteriocin	*L. casei* L26	*E. coli* O111, *L. monocytogenes*	Mouse	Su et al. (2007)
Unnamed bacteriocin	*L. johnsonii* La1	*Helicobacter pylori*	Children and adults	Gotteland (2003), Cruchet et al. (2003)
Salivaricin	*S. salivarius* CRL1328	*Enterococcus* spp., *Neisseria gonorrhoeae*	In vitro	Juarez Tomás et al. (2002)
Salivaricin A & B	*S. salivarius* K12	*Streptococcus sobrinus*, *S. mutans*	Children and adults	Burton et al. (2006b), Dierksen et al. (2007)
Salivaricin B	*S. salivarius* K12	*Micrococcus luteus; S. anginosis; Eubacterium saburreum*	Humans	Burton et al. (2006a)
Salivaricin	*S. salivarius* K12	*S. pyogenes*	Children	Walls et al. (2003)
ESL5	*E. faecalis* SL-5	*Propionibacterium acnes*	In vitro and human	Kang et al. (2009)
Diffocin	*C. difficile CD4*	*C. difficile*	In vitro and mice	Gebhart et al. (2015), Kåhrström (2015)
Subtilosin	*B. subtilis*	*Gardnerella vaginalis*, *L. monocytogenes*, *S. agalactiae*	In epivaginal	Sutyak et al. (2008a, b)
Laterosporulin10	*B. laterosporus* SKDU10	*S. aureus*, *M. smegmatis*	In vitro and macrophages	Baindara et al. (2016)
NVB333 lanthipeptide	*Actinoplanes liguriae* NCIMB41362	*S.aureus*	In vitro and mice	Boakes et al. (2016)
Pediocin PA-1	*P. acidilactici*	*L. monocytogenes*	Mouse	Dabour et al. (2009)
Bacteriocins ST651ea, ST7119ea, and ST7319ea	*E. faecium* ST651ea, ST7119ea, and ST7319ea	*L. monocytogenes* and vancomycin-resistant enterococci	Simulated gastrointestinal tract	Fugaban et al. (2021a)

Bacteriocin	Producer	Antiviral agents		
		Target virus	Model	References
Bacteriocin-like inhibitory substances	*Enterococcus faecium* CM019	Severe acute respiratory syndrome coronavirus 2 (SARS-CoV-2)	Vero-E6 cells	Bahy et al. (2023)
Labyrinthopeptin A1	*Actinomadura namibiensis* DSM 6313	Human immunodeficiency virus (HIV), Herpes simplex virus (HSV), dengue virus, and Zika virus	In vitro	Férir et al. (2014)
Mundticin ST4SA	*E. mundtii* ST4V	HSV-1, HSV-2, Measles virus, and poliovirus	In vitro	Todorov et al. (2005)
Subtilosin	*B. subtilis*	HSV-1 and HSV-2	In vitro	Quintana et al. (2014)
Subtilosin	*B. amyloliquefaciens*	HSV-1	In vitro	Torres et al. (2013)
Enterocin AAR-74	*E. faecalis*	Coliphage HSA	In vitro	Qureshi et al. (2006)
Enterocin B	*E. faecium* L3	Influenza A virus subtype H3N2, H1N1	In vitro and mouse	Ermolenko et al. (2019)
Enterocin CRL35	*E. faecium* CRL3	HSV-1 and HSV-2	In vitro	Wachsman et al. (2003)
Enterocin CRL35	*E. mundtii*	Herpesviruses	Vero and BHK-21 cells	Wachsman et al. (1999)
Enterocin ST5Ha	*E. faecium* ST5Ha	HSV-1	In vitro	Todorov et al. (2010)
Enterocin AAR-71	*E. faecalis*	Coliphage HSA	In vitro	Qureshi et al. (2006)
Unnamed bacteriocins	*L. lactis* subsp. Lactis and* E. durans*	HSV-1 and poliovirus (PV-1)	Vero cells	Cavicchioli et al. (2018)
Unnamed bacteriocins	*L. delbrueckii*	Influenza viruses (H7N7 and H7N1)	In vitro	Serkedjieva et al. (2000)
Erwiniocin NA4	*Erwinia carotovora* NA4	Coliphage HSA	In vitro	Qureshi et al. (2006)
Staphylococcin 188	*S. aureus* AB188	New castle disease virus (NCDV), poliovirus	In vitro and in vivo	Saeed et al. (2007)
Erwinaocin NA4	*E. carotovora* NA4	Coliphage	In vitro	Dey et al. (2021)

Bacteriocin	Producer	Anticancers		
		Target cancer cell lines	Model	References
Laterosporulin 10	*B. laterosporus* SKDU10	MCF-7, HEK293T, HT1080, HeLa and H1299 cells	In vitro	Baindara et al. (2017)
Microcin E492	*K. pneumoniae*	Human cell lines	In vitro	Hetz et al. (2002)
Microcin E492	*K. pneumoniae*	Human colorectal cancer cells	In vivo SW480 and SW620 zebrafish xenograft	Varas et al. (2020)
Nisin	*L. lactis*	Human asterocytoma cell line (SW1088), head and neck squamous cell carcinoma (HNSCC)	In vitro	Joo et al. (2012), Zainodini et al. (2018)
Nisin	*L. lactis*	Colon cancer cell line	In vitro	Ahmadi et al. (2017)
Nisin A	*L. lactis*	Head and neck squamous cell carcinoma (HNSCC)	In vitro	Shin et al. (2016)
Plantaricin P1053	*L. plantarum* PBS067	Cancerogenic epithelial intestinal cell lines	In vitro	De Giani et al. (2019)
Plantaricin A	*L. plantarum* C11	GH4, Reh, Jurkat, PC12, N2A	In vitro	Sand et al. (2013)
Enterocin LNS18	*Enterococcus thailandicus*	HepG2 cell lines	In vitro	Al-Madboly et al. (2020)
Pediocin K2a2-3	*P. acidilactici* K2a2-3	Human colon adenocarcinoma (HT29) and human cervical carcinoma (HeLa) cells	In vitro	Villarante et al. (2011)
Pediocin CP2	*P. acidilactici* CP2 MTCC501	HeLa, MCF-7, HepG2, murine myeloma (Sp2/0-Ag 14)	In vitro	Kumar (2012)
Duramycin	*S. cinnamoneus*	AsPC-1, Caco-2, Colo320, CT116, JJN3, Lovo, MCF-7, (Rodrigues et al. 2019) MDA-B-231, MIA PaCa-2	In vitro	Broughton et al. (2016)
Pep27anal2	*S. pneumoniae*	Jurkat, HL-60, AML-2, MCF-7, SNU-601	In vitro	Lee et al. (2005), Sung et al. (2007)
Bovicin HC5	*S. bovis* HC5	MCF-7, HepG2 mammalian cell lines	In vitro	Mantovani et al. (2002), Paiva et al. (2012)
p28	*Pseudomonas aeruginosa* PAO1	MCF-7, HCT-116, UISO-MEL-23, MNE-MB-231, p53wt (Mel-29), U87, LN229	In vitro	Yamada et al. (2009), Mehta et al. (2011)
Pyocin S2	*P. aeruginosa* 42A	HepG2, Im9, murine tumor (mKS-A TU-7), human fetal foreskin fibroblast (HFFF)	In vitro	Abdi-Ali et al. (2004)
Colicin E3	*E. coli*	P388, HeLa, HS913T	In vitro	Kohoutova et al. (2014)
Sungsanpin	*Streptomyces* spp.	Human lung cancer cell line A549	In vitro	Um et al. (2013)
Chaxapeptin	*S. leeuwenhoekii* C58	Human lung cancer cell line A549	In vitro	Elsayed et al. (2015)
Thiostrepton	*S. aureus*	Breast cancer cell lines, endometriosis	Rat	Kwok et al. (2008), Jin et al. (2019), Kongsema et al. (2019)

Bacteriocin	Producer	Food preservation, safety, and quality		
		Target microorganism	Food/model	References
Colicins (GRN 676, GRN 593)	*E. coli*	*E. coli*, *P. aeruginosa*, *Salmonella* spp.	Meat, fruits, and vegetables	Hahn-Löbmann et al. (2019)
Sakacin P	*L. sakei*	*L. monocytogenes*	Beef and salmon	Teneva-Angelova et al. (2018)
Sakacin	*Lactobacillus sakei* subsp. *sakei* 2a	*L. monocytogenes*	Cheese	Martinez et al. (2015)
Salmocins	*Salmonella* spp.	*S. enterica*	Red meat	Schneider et al. (2018)
Divergicin M35	*Carnobacterium divergens* M35	*L. monocytogenes*	Smoked fish	Benabbou et al. (2020)
Lactocin 705, Lactocin AL705	*Lactobacillus curvatus* CRL705	*B. thermosphacta*,* L. innocua*	Vacuum-packed meat	Castellano and Vignolo (2006)
Lactoccin BZ	*Lactococus lactis*	*L. innocua*	fresh beef	Yıldırım et al. (2016)
Enterocin K2B1	*E. faecalis* K2B1	Foodborne pathogens	Dairy products	Alang et al. (2020)
Enterocin AS-48	*Enterococcus faecalis*	Endogenous staphylococci	Sardines	Ananou et al. (2014)
Enterocin LD3 and Plantaricin LD4	*E. faecium* LD3 *and L. plantarum* LD4	*S. aureus* subsp. *aureus* ATCC25923, *Salmonella enterica* subsp*. enterica serovar* Typhimurium ATCC13311, *Proteus mirabilis* ATCC43071, *P. aeruginosa* ATCC27853, and *E. coli* ATCC25922	In vitro	Sheoran and Tiwari (2021)
Aureocin A70	*S. aureus* A70	*L. monocytogenes*	Dairy products	Carlin Fagundes et al. (2016)
Psicolin 126, carnocyclin A	*Carnobacterium maltoaromaticum*	*L. monocytogenes*	Ready-to-eat meat products	Liu et al. (2008)
Variacin	*Kocuria varians* NCC 1482	*B. cereus*	Dairy food	O’Mahony et al. (2001)
Lacticin 481	*L. lactis* L3A21M1	*L. monocytogenes*	Fresh cheese	Ribeiro et al. (2016)
Lacticin 3147	*L. lactis* subsp. *lactis* DPC3147	*L. monocytogenes*	Cottage cheese and yogurt	Morgan et al. (2001)
Reuterin	*L. reuteri* INIA PRO 137	*L. monocytogenes* and *S. aureus*	skim milk	Arqués et al. (2011)
Gassericins A and T	*L. gasseri* LA39 and LA158	*B. cereus*	Custard cream	Arakawa et al. (2009)
Bovicin HC5	*S. bovis* HC5	*Clostridium tyrobutyricum*	Mango pulp	de Carvalho et al. (2007)
Ent35-MccV	*E. coli* BL21	*E. coli* and* L. monocytogenes*	Skim milk	Acuña et al. (2015)
Bacteriocin GP1	*L. rhamnosus* GP1	*Staphylococcus* spp., *Aeromonas* spp., *Lactobacillus* spp., *Pseudomonas* spp., *Vibrio* spp.	Fish	Sarika et al. (2019)
Bacteriocins ST3522BG and ST3633BG	*P. acidilactici* ST3522BG and *P. pentosaceus* ST3633BG	*Listeria* spp.	Silage fermentation models system	Fugaban et al. (2021b)
Bacteriocin BM1829	*Companilactobacillus crustorum* MN047	*E. coli* and *S. aureus*	Beef	Yan et al. (2021)
Bacteriocin Sak-59	*L. sakei* B-RKM 0559	*L. monocytogenes*, *S. aureus*, and pathogenic strains of *Serratia marcescens* and* E. coli*	Meat spoilage bacteria	Abitayeva et al. (2021)
Bacteriocins ST20Kc and ST41Kc	*E. faecium* ST20Kc and ST41Kc	*L. monocytogenes* and vancomycin-resistant enterococci	Kimchi	Valledor et al. (2022)
Bacteriocin 32Y	*L. curvatus*	*L. monocytogenes*	Pork and beef	Gálvez et al. (2007)
Bacteriocin RSQ04	*L. lactis* CGMCC20699	*L. monocytogenes*	Model food system	Xiang et al. (2022)
Bacteriocin OS1	*E. hirae* OS1	*Listeria* spp.	In vitro	Siragusa (1992)
Pyocin QDD1	*P. aeruginosa* QDD1	*S. aureus* and* B. cereus*	In vitro	Doshi et al. (2022)
Nisin (Nisaplin^®^)	*L. lactis*	*S. aureus*	Minas frescal cheese	Felicio et al. (2015)
Nisin Z	*L. lactis* W8	*Enterococcus italicus*, *E. mundtii*, *E. faecalis*, *B. thuringiensis*, *B. cereus*, *L. paracasei*, *Acinetobacter* spp., *Pseudomonas fluorescens* and* Enterobacter aerogenes*	Skim and whole-fat milk	Mitra et al. (2011)
Nisin Z and A and lacticin 481	*L. lactis*	*L. monocytogenes*	Cottage cheese	Dal Bello et al. (2012)
Nisin	*L. lactis* N5764	*S. aureus* and *L. monocytogenes*	Cow milk	Alves et al. (2016)
Micrococcin P1	*S. equorum* WS 2733	*L. monocytogenes*	Soft cheese	Carnio et al. (2000)
AMA-K, Leucocin K7	*L. plantarum* AMA-K	*K. pneumoniae*, *Listeria* spp., *Enterococcus* spp.,* E. coli*	Amasi (fermented milk product)	Todorov (2008)

Bacteriocin	Producer	Antimicrobial food packaging		
		Target microorganism	Food/model	References
Nisin	*L. lactis*	*S. aureus*, *L. monocytogenes*	Cellulose films + minimally processed mangoes	Barbosa et al. (2013)
Nisin Z	*L. lactis* subsp. lactis I8-7-3	*Salmonella typhimurium*, *S. enteriditis*, *S. aureus*, *L. monocytogenes*, *E. coli*	Pullulan films + fresh and ready to eat muscle foods	Pattanayaiying et al. (2015)
Nisin	*L. lactis*	*E. coli* O157:H7, *Salmonella* spp.	Stainless steel	Phongphakdee and Nitisinprasert (2015)
Nisin	*L. lactis*	*Micrococcus luteus* ATCC 10240	Ethylene-*co*-vinyl acetate (EVA) film	(Scaffaro et al. 2011)
Nisin	*L. lactis*	*S. aureus* and* E. coli*	Poly(vinyl alcohol) films	Hrabalikova et al. (2016)
Nisin	*L. lactis*	*E. coli* O157:H7, *Salmonella*, and* L. monocytogenes*	Fresh cut cantaloupe/rind	Ukuku et al. (2015)
Nisin	*L. lactis*	*L. monocytogenes*	Starch/halloysite/nanocomposite films + soft cheese	Meira et al. (2016)
Nisin and lacticin 3147	L. lactis subsp. lactis HP	*L. lactis* subsp. *lactis*, *S. aureus*, and* L. innocua*	Polyamide and polyethylene pouches + cheese	Scannell et al. (2000)
Sakacin A	*L. sakei*	*L. monocytogenes*	Polyethylene coated paper sheets + meat	Barbiroli et al. (2017)
Curvacin A	*L. sakei* CRL1862	*L. monocytogenes*	Stainless steel Polytetrafluoroethylene surfaces (PTFE)	Pérez-Ibarreche et al. (2016)
Lacticin	*L. lactis*	*L. helveticus* and *Brochothrix thermosphacta*	Polyethylene based plastic film + meat	Siragusa et al. (1999)
Divergicin M35	*Carnobacterium divergens M35*	*L. monocytogenes*	Chitosan film + smoked fish	Benabbou et al. (2020)
Bacteriocin 7293	*Weissella hellenica* BCC 729	Gram-positive and Gram-negative food borne pathogens	PLA/SP biocomposite film + pangasius fish fillets	Woraprayote et al. (2018)
Plantaricin BM-1	*L. plantarum* BM-1	*L. monocytogenes*	Polyethylene	Zhang et al. (2017)
Enterocin B3A-B3B	*E. faecalis* B3A-B3B	*L. monocytogenes*	Stainless steel	Al-Seraih et al. (2017)
Pediocin	*P. acidilactici*	*L. monocytogenes*	Plastic bags and cellulose casings + meat	Ming et al. (1997)

Bacteriocin	Producer	Antibiofilm and sanitizers		
		Target microorganism/biofilm former	Model	References
Gallidermin	*S. gallinarum*	*S. aureus* and *S. epidermidis*	Medical implants	Saising et al. (2012)
Nisin	*L. lactis*	*L. monocytogenes* 4032	Stainless steel and polypropylen	Saá Ibusquiza et al. (2011)
Nisin, enterocin DD14, colistin combination	*L. lactis* and *E. faecalis* 14	*E. coli* CIP54127, *E. coli* 184 (mcr-1+), and *E. col*i (mcr-1)	In vitro	Al Atya et al. (2016a)
Lacticin 3147	*L. lactis*	*S. mutans*	In vitro oral biofilm model	Corbin et al. (2011)
Bacteriocins 4356 and 8014	*L. acidophilus* ATCC 4356 and *L. plantarum* ATCC 8014	*Serratia marcescens*	In vitro	Vahedi Shahandashti et al. (2016)
Hyicin 4244	*Staphylococcus hyicus* 4244	14 *Staphylococcus* strains from human infections or bovine mastitis	In vitro	Duarte et al. (2018)
Licheniocin 50.2	*L. lactis* subsp. lactis biovar. diacetylactis BGBU1-4	*L. monocytogenes*, coagulase-negative staphylococci	In vitro	Cirkovic et al. (2016)
Sonorensin	*Bacillus sonorensis* MT93	*L. monocytogene*s and *S. aureus*	Polyethylene film coated meat and tomatoes	Chopra et al. (2015)
Enterocin AS-48	*E. faecalis* A-48-32	*L. monocytogenes*	In vitro	Caballero Gómez et al. (2013)
Enterocin AS-48 with benzalkonium chloride, polyhexamethylene guanidium chloride and triclosan	*E. faecalis* A-48-32	MRSA and MSSA	In vitro	Caballero Gómez et al. (2013)
Enterocin AS-48 with biocides	*E. faecalis* A-48-32	*L. monocytogenes*	In vitro	Gómez et al. (2012)
Enterocin DD93, DD28	*E. faecalis* DD28 and *E. faecalis* DD93	MRSA	In vitro, stainless steel, and glace devices	Al Atya et al. (2016b)
Enterocin B3A-B3B	*E. faecalis* B3A-B3B	*L. monocytogenes*	Stainless steel	Al-Seraih et al. (2017)
Unnamed bacteriocin	*L. fermentum 97*	*S. epidermidis*, enterotoxigenic enterobacteria	In vitro	Rybalchenko et al. (2015)
Unnamed bacteriocin	*Citrobacter freundii*	*Citrobacter*, *K. pneumoniae*, *E. coli*	In vitro	Shanks et al. (2012)
Curvacin A	*L. sakei* CRL1862	*L. monocytogenes*	Stainless steel, polytetrafluoroethylene surfaces (PTFE)	Pérez-Ibarreche et al. (2016)

Bacteriocin	Producer	Aquaculture/aquatic product		
		Target microorganism	Application/model	References
CAMT2	*Bacillus amyloliquefaciens* ZJHD3-06	*L. monocytogenes*, *S. aureus*	*Epinephelus areolatus*	An et al. (2015)
Coagulin L1208	*B. coagulans* L1208	*E. coli*, *Shewanella putrefaciens*,* S. aureus*	*Pseudosciaena croce*	Fu et al. (2018)
Mundticin KS	*E. mundtii* Tw56	*P. aeruginosa*,* S. putrefaciens*	*Odontesthes platensis*	Schelegueda et al. (2015)
BacALP7	*E. faecium*	*L. monocytogenes*	Shellfish	Pinto et al. (2009)
Nisin Z	*L. lactis* ssp. Lactis	*Streptococcus iniae*	*Oxyeleotris lineolata*	Wright (2017)
Nisin Z	*L. lactis* TW34	*L. garvieae*	*Odontesthes platensis*	Sequeiros et al. (2015)
Nisin	*L. lactis*	*L. monocytogenes*	*Litopenaeus vannamei*	Zhao et al. (2020)
Plantaricin FGC-12	*L. plantarum* FGC-12	*V. parahaemolyticus*	Golden carp	Chen et al. (2019)
Weissellicin 110	*Weissella cibaria*	*L. sakei* JCM 1157	Plaa-Som, a Fermented Fish Product	Srionnual et al. (2007)
Enterocin MC13	*E. faecium* MC13	*L. monocytogenes*, *V. parahaemolyticus*, and *V. vulnificus*	*Mugil cephalus*	Satish Kumar et al. (2011)
Pentocin JL-1	*L. pentosus*	*S. aureus*	*Chiloscyllim punctatum*	Jiang et al. (2017)
PE-ZYB1	*P. Pentosaceus* Zy-B	*L. monocytogenes*	*Mimachlamys nobilis*	Zhang et al. (2020)
Unnamed bacteriocin	*P. acidilactici*	*L. monocytogenes*	*Tilapia* sp., Catla catla, Cyprinus carpio	Sudarsanan and Thangappan (2017)
Bacteriocin 7293	*W. hellenica* BCC 7293	*L. monocytogenes*, *S. aureus*, *A. hydrophila*, *E. coli*, *P. aeruginosa*, *S.* Typhimurium	*Pangasius bocourti*	Woraprayote et al. (2018)
Bacteriocin KTH0-1S	*L. lactis* KTH0-1S	*S. aureus*	Fermented shrimp	Saelao et al. (2017)
Bacteriocin PSY2	*L. lactis* strain PSY2	Spoilage Gram-positive and Gram-negative bacteria	Perch	Sarika et al. (2012)
Bacteriocin CN-25	*E. faecium* CN-25	*L. monocytogenes*	Fermented fish roe	du Toit et al. (2000)

Bacteriocin	Producer	Plant diseases		
		Target phytopathogen	Application/model	References
Gluconacin	*Gluconacetobacter diazotrophicus* strain PAL5	*Xanthomonas axonopodis* pv*. vasculorum*, *Acidovorax avenae* subsp*. avenae*, *Pseudomonas syringae* pv*. syringae*, *Xanthomonas vasicola* pv*. vasculorum*	In vitro	Oliveira et al. (2018)
Amylocyclicin	*B. amyloliquefaciens* FZB42	*Ralstonia solanacearum* and* X. campestris*	In vitro	Scholz et al. (2014)
Enterocin UNAD 046	*E. faecalis*	*Botryodiplodia theobromae*, *Aspergillus niger*, *Pythium ultimum*, *Penicillium expansum*, and* Fusarium oxysporum*	In vitro	David and Onifade (2018)
Putidacin L1 (PL1)	*Pseudomonas putida*	*P. syringae*	In vitro	Rooney et al. (2020)
Tailocins	*Pseudomonas fluorescens* SF4c	*X. vesicatoria* Xcv Bv5-4a	Tomato fruits	Príncipe et al. (2018)
Syringacin M	*Pseudomonas syringae* pv. *tomato* DC3000	*P. syringae*	*Arabidopsis* and tomato plants	Li et al. (2020)
Plantazolicin	*B. amyloliquefaciens* subsp. Plantarum FZB42	*B. anthracis* and *nematodes*	Plant roots	Chowdhury et al. (2015)
Carocin D	*P. carotovorum* subsp. Carotovorum	*P. carotovorum* subsp. Carotovorum	In vitro	Grinter et al. (2012)
Kenyacin 404, Entomocin 420, Tolworthcin 524, Morricin 269, Kurstacin 287	*B. thurigiensis*	*F. oxysporum*, *Rhizopus* sp., *Mucor rouxi*, *Trichoderma* spp*.*, *A. nodulans*, *F. graminis*,	In vitro	Salazar-Marroquín et al. (2016)
BLIS RC-2	*B. amyloliquefaciens* RC-2	*X. campestris pv.* Campestris,*C. dematium*, *R. necatrix*, *P. oryzae*,* A. tumefaciens*	In vitro	Abriouel et al. (2011)
Bacteriocin LlpA	*Pseudomonas* sp. strain BW11M1	*P. fluorescens* Pf-5, *P. tolaasii*	In vitro	Parret et al. (2005)
Unnamed bacteriocin	*B. gladioli*	*Tatumella ptyseos*	In vitro and in planta	Marín-Cevada et al. (2012)
Unnamed bacteriocin	*P. syringae* pv. Ciccaronei	*P. syringae* subsp. Savastanoi	In vitro and in planta	Lavermicocca et al. (2002)
BL8	*B. thuringiensis* subsp. Tochigiensis HD868	*Cryphonectria parasitica*, *F. oxysporum*, *Penicillium digitatum*, *A. niger*, *A. fumigatus*,* A. flavus*	In vitro	Subramanian and Smith (2015)

### Modulation of microbiomes

The microbiota is crucial and necessary for maintaining homeostasis, the host defense system, disease prevention, and overall health and well-being. The composition and diversity of the microbiota vary depending on localized regions (e.g., oral, nasal, respiratory, gut, and skin) and consist of highly diverse and complex communities with specialized autochthonous bacteria (Berg et al. 2020; Anjana 2022; Baquero et al. 2019; Zheng et al. 2023; Ormaasen et al. 2023; Reuben et al. 2023; Pérez-Cobas et al. 2023; Ferraz 2023). Dysbiosis of the microbiota often leads to physiological dysfunction, dysregulation, and diseases (Hou et al. 2022). Numerous studies have highlighted the indiscriminate impact of antibiotics on the microbiota, resulting in dysbiosis and perturbations of microbial composition and diversity that predispose the host to metabolic and immune system disorders (Francino 2015; Sanchez-Rodriguez et al. 2020; Hou et al. 2022). Unlike antibiotics, bacteriocins have a narrow spectrum of activity, are highly specific, and can inhibit pathogens without disrupting host-microbiota homeostasis or causing detrimental effects. Bacteriocins that can promote beneficial shifts in the abundance, composition, and diversity of the microbiota may provide sustainable and valuable microbiome-based solutions for the treatment of infectious and non-infectious microbiome-related diseases resulting from microbiota dysbiosis.

Furthermore, bacteriocin production by most bacteria can be seen as a strategy to modulate the microbiome (Pu et al. 2022; O’Reilly et al. 2023; Ríos Colombo et al. 2023; Rani and Tiwari 2023; Puls et al. 2024). Bacteriocins can either prevent invasion by allochthonous bacteria (competitors or pathogens) or stimulate the immune system to prevent oxidative stress and inflammation (Dahiya et al. 2017; Bäuerl et al. 2017; Heilbronner et al. 2021; Rani and Tiwari 2023; Puls et al. 2024). In another instance, bacteriocin-producing bacteria can invade and colonize communities predominantly populated by susceptible strains (Riley and Gordon 1999; Heilbronner et al. 2021). Bacterial interactions within the microbiota are characterized by both competition (antagonism) and cooperation (mutualism), which require a delicate balance for overall microbiota functioning and cohesion (Heilbronner et al. 2021; Pérez-Cobas et al. 2023). However, the mechanisms regulating the integration and modulation of bacteriocins in this complex multifactorial meshwork remain a black box.

Although the roles of bacteriocins in microbiome modulation and the maintenance of homeostasis and host health are limited, extensive metagenomic analysis substantially revealed the omnipresence of bacteriocin biosynthetic gene clusters across human microbiomes (Donia et al. 2014; Aleti et al. 2019; Naimi et al. 2022). In a study, several bacteriocins, including garvicin ML (GarML), plantaricins EF and JK (plantaricins), enterocins P, Q, and L50 (enterocins), pediocin PA-1 (PedPA-1), and sakacin A (SakA) were reported to beneficially modulate the gut microbiota in mice (Umu et al. 2016). While these bacteriocins differ greatly in terms of physicochemical properties and inhibition spectrum, their administration had a favorable impact on the microbiota, resulting in changes at the taxonomic level, increased abundance of LAB, and a decrease in Enterococcaceae, clostridia, and staphylococci. Recent studies showed that nisin, lacticin 3147, pediocin PA1, and bactofencin A separately modulated gut microbiota, resulting in subtle and beneficial alterations in pigs, Simplified Human Intestinal Microbiota (SIHUMI), and simulated colon models (Ríos Colombo et al. 2023; O’Reilly et al. 2023; Pu et al. 2022; Guinane et al. 2016). Bactofencin A increased the relative abundances of *Bifidobacterium* and *Streptococcus* while lowering the abundances of *Blautia* and *Clostridium* spp. (Arboleya et al. 2016; Sun et al. 2020). *Bifidobacterium* spp. are considered important microbes in healthy microbiota and are associated with probiotic properties. Mice fed with bacteriocin-producing *L. salivarius* UCC118 for eight weeks showed changes in gut microbiota compared to those fed with non-bacteriocin-producing variants (Murphy et al. 2013). Treatment with bacteriocin-producing *L. salivarius* UCC118 significantly increased *Proteobacteria* and *Bacteroides* while decreasing Actinobacteria. Similarly, the assessment of *L. salivarius* bacteriocin, bactofencin A, in a simulated gut microbiota system showed significant microbiota modulation in both the bactofencin A-producing strain and bactofencin A treatments compared with the non-bactofencin A producing mutant (Guinane et al. 2016). Bacteriocin production subtly changes the community structure of the gut microbiota at the taxonomic level, maintaining a beneficial and desirable microbiota (Guinane et al. 2016; Garcia-Gutierrez et al. 2019; O’Connor et al. 2020). In the same manner, Naimi et al. (2022) recently reported the subtle beneficial modulatory effect of Microcin J25 (MccJ25) or reuterin on the overall colon microbiota diversity and metabolome of swine.

Within the oral cavity, some strains of *S. mutans* produce bacteriocins called mutacins which modulate the oral microbiome by inhibiting phylogenetically related plaque-forming strains (Gillor et al. 2008). There is a positive correlation between the production of bacteriocins by *S. mutans* and their ability to colonize the oral cavity (Hillman et al. 1987, 2000). *S. salivarius* K12, a commensal of the oral cavity often produces bacteriocins called salivaricins A and B. The presence of *S. salivarius* K12 which produces salivaricins A and B has been shown to modulate the oral and throat microbiomes, preventing the invasion of oral pathogens such as *S. pyogenes* and reducing throat infections (Brook 2005; Horz et al. 2007). Similarly, the consumption of milk containing a strain of *S. salivarius* 20P5, which produces salivaricin A, positively modulates the oral microbiota of children by significantly increasing the production and antagonistic activity of salivaricin A and providing immunity against *S. pyogenes* infection (Walls et al. 2003). Bacteriocin-producing *Lactobacillus* spp. including *L. gasseri*, *L. crispatus*, *L. jensenii*, and *L. iners*, are dominant in the vagina microbiota of healthy women (Vásquez et al. 2002; Pendharkar et al. 2023). In contrast, women with bacterial vaginosis have a distinct vaginal microbiota characterized predominantly by *Mycoplasma hominis*, *Gardnerella vaginalis*, *Bacteroides*, *Mobiluncus*, *Peptostreptococcus*, and *Prevotella* spp., along with lower densities of lactobacilli (O’Brien 2005; Falagas et al. 2007; Turovskiy et al. 2009). The vaginal microbiota is often modulated by bacteriocin-producing lactobacilli, which typically antagonize pathogens, especially *G. vaginalis* and *Candida* spp. (Kaewsrichan et al. 2006; Günther et al. 2022).

The skin microbiome consists of a highly diverse array of microorganisms involved in complex but balanced multifactorial interactions with the host and external environment (Carmona-Cruz et al. 2022; Nicholas-Haizelden et al. 2023; Glatthardt et al. 2024). Any imbalance (dysbiosis) in the structure and composition of the skin microbiota often results in skin infections/diseases such as acne, impetigo, atopic dermatitis, and psoriasis (Grice 2014; O’Sullivan et al. 2019; Carmona-Cruz et al. 2022; Richter and Wohlrab 2023; Sato et al. 2023; Puls et al. 2024). Bacteriocins have been used to selectively modulate and restore the skin microbial balance (eubiosis) in situations of dysbiosis caused by pathogen colonization and environmental perturbation (O’Sullivan et al. 2019; Ovchinnikov et al. 2020; Soltani et al. 2022b; Alessandrini et al. 2023; Jaumaux et al. 2023). Lugdunin, a cyclic peptide bacteriocin facilitates the restoration of skin microbial balance while inhibiting different etiological agents of skin infections, especially MRSA and other Gram-positive bacteria (Bitschar et al. 2019; Krauss et al. 2020; Barber and Zhang 2021; Bier and Schittek 2021). Lugdunin is believed to exert microbiome modulatory activity by stimulating the expression of different cutaneous antimicrobial peptides and recruiting phagocytic neutrophils and monocytes (Bitschar et al. 2019; Krauss et al. 2020; Saur et al. 2021; Hirsch et al. 2024). Lugdunin also inhibits colonizing skin pathogens by disrupting the transmembrane pH gradient, which likely leads to protein denaturation and a reduction in proton motive force, obstructing cellular respiration (Krulwich et al. 2011; Farha et al. 2013; Barber and Zhang 2021). Similarly, two recently discovered bacteriocins, cerein B4080 and cerein 7B, reportedly enhance skin microbiome eubiosis by selectively promoting the growth of skin commensals while inhibiting pathogens (Jaumaux et al. 2023). By preserving skin commensals through competitive exclusion/inhibition of pathogens, bacteriocins could beneficially modulate the skin microbiome while limiting the emergence and spread of superbugs within the skin ecosystem, thereby reducing skin infections (Meade et al. 2020; Soltani et al. 2022b; Jaumaux et al. 2023). Other bacteriocins that show high potential for application in skin microbiome modulation include garvicin KS, nisin Z, bactofencin A, pediocin PA-1, subtilosin, microcin J25, micrococcin P1, subtilin, bacteriocin A37, and reuterin (Joseph et al. 2013; O’Sullivan et al. 2019; Ovchinnikov et al. 2020; Heilbronner et al. 2021; Soltani et al. 2022b; Alessandrini et al. 2023; Puls et al. 2024). There is a need to further explore the mechanisms of activity and pharmacological benefits of promising skin-relevant bacteriocins for their suitability in clinical application and commercialization.

Bacteriocins have also been used to modulate food microbiota to improve organoleptic properties, quality, and microbiological safety. The growing knowledge of the structure and function of food microbiota now influences their modulation towards desirable functions and beneficial outcomes. Food microbiota are often modulated through the regulation of abiotic factors or by using specific microorganisms and/or their products, such as bacteriocins (And and Hoover 2003; Walsh et al. 2023). The latter involves the use of various forms of bacteriocins, whether purified or semi-purified, and/or bacteriocin-producing strains to modulate food microbiota (O’Sullivan et al. 2003; Ramu et al. 2015; Silva et al. 2018). It has been demonstrated that the microbiota of fermented foods (e.g., cheese and kefir) can be modulated, making them useful models for shaping food microbiota (Wolfe et al. 2014; Bonham et al. 2017; Wolfe 2018; Blasche et al. 2021; Walsh et al. 2023). The application of bacteriocins or bacteriocin-producing strains as starter or protective cultures in dairy products can confer numerous advantages during food processing. They can modulate the food microbiota by accelerating ripening, as is the case with cheese (Ávila et al. 2005; Martinez et al. 2015), or reduce the growth of adventitious non-starter lactic acid bacteria (NSLAB) and other non-starter microbiota in fermented foods (Oumer et al. 2001; O’Sullivan et al. 2003), or inhibit invasion by environmental or spoilage organisms (Muñoz et al. 2004, 2007), or significantly reduce the growth of foodborne pathogens (Carnio et al. 2000; Aspri et al. 2017; Kondrotiene et al. 2018), or accelerate enzyme release and activities (O’Sullivan et al. 2003), or enhance fermentation (Oumer et al. 2001). Additionally, bacteriocin production has been detected in LAB bacteria recovered from wine during malolactic fermentation, especially among *L. plantarum* strains (Navarro et al. 2000; Rojo-Bezares et al. 2008; Díez et al. 2012). During vinification, bacteriocin production could be an important characteristic to consider when selecting LAB as starters for malolactic fermentation. Furthermore, bacteriocins produced by LAB have significant potential for use as biocontrol agents against foodborne and spoilage organisms as well as biopreservatives throughout the enological processes (Díez et al. 2012; Dündar 2016; Fernández-Pérez et al. 2018).

### Medical and pharmaceutical applications

The emergence and spread of infectious diseases, especially those caused by antimicrobial-resistant pathogens, and the increasing morbidity and mortality due to non-communicable diseases like diabetes and cancer pose major threats to global health (PAHO/WHO 2019; WHO 2021). Due to their high antimicrobial activity against a wide range of pathogens, safety, biocompatibility, unique mechanisms of action, biodegradability, high specificity, and nanomolar range, bacteriocins exert desirable heterogeneous traits relevant for medical application (Naveen and Kalaivani 2018; Meade et al. 2020; Le et al. 2021, 2023; Reinseth et al. 2024; Rossi et al. 2024). The potential of bacteriocins in medicine has been demonstrated through various in vitro, ex vivo, and in vivo experiments, with some undergoing clinical evaluation. However, concerns have risen regarding solubility, stability, bioavailability, sensitivity to proteolytic enzymes, high cost, and the challenges of large-scale purification and production for general use, which often limit the direct use of bacteriocins in clinical studies and hinder their industrial production and commercialization (Böttger et al. 2017; Mathur et al. 2018; Hols et al. 2019; Soltani et al. 2021a). Nevertheless, due to the unique and diverse medical potentials exhibited by bacteriocins, further investigations involving cutting-edge bioengineering techniques can be conducted to address these concerns and improve their properties and large-scale production for general medical use.

#### Inhibition of pathogens: viable alternatives to antibiotics

Since the discovery of antibiotics, they have played a significant role in the prevention and treatment of animal and human diseases. However, the emergence and increasing spread of multi- and extensive-drug-resistant superbugs necessitate the urgent use of novel, suitable, and sustainable strategies for infection control, treatment, and addressing AMR concerns. Bacteriocins show great promise as sustainable alternatives to currently available antibiotics. Numerous studies have described the unique mechanisms of action and potency of different bacteriocins against a broad range of superbugs (Bastos et al. 2009, 2015; Svetoch et al. 2009; Ahmad et al. 2017; Goodarzi et al. 2020; Ovchinnikov et al. 2021; Benítez-Chao et al. 2021; Sharma et al. 2022; Soltani et al. 2022a; Barman et al. 2023; Ghapanvari et al. 2022; Bahy et al. 2023; Ibraheim et al. 2023; Wolden et al. 2023; Reinseth et al. 2024). Over the years, many studies have reported the antimicrobial properties of various bacteriocins against clinically important pathogens responsible for respiratory tract, nosocomial, dental, skin, and gastrointestinal tract infections. Bacteriocins have also been shown to have inhibitory effects on multidrug-resistant pathogens including *C. difficile*, vancomycin-resistant *Enterococcus* (VRE), methicillin-resistant *S. aureus* (MRSA), *Klebsiella pneumoniae*, *Pseudomonas aeruginosa*, *Haemophilus influenza*, *Listeria* spp., *Salmonella* spp., *Enterobacter* spp., *Acinetobacter* spp. and others (Oman and van der Donk 2009; Lay et al. 2016; Hanchi et al. 2017; Yu et al. 2019; Velázquez-Suárez et al. 2021; Ghapanvari et al. 2022; Bahy et al. 2023; Le et al. 2023; Alattar et al. 2024; Mu et al. 2024; Reinseth et al. 2024). Recently, Ying et al. (2024) and Wolden et al. (2023) separately identified novel bacteriocins, bacteriocin XJS01 and romsacin (produced by *Lactobacillus salivarius* and *Staphylococcus haemolyticus*) which showed broad-spectrum activity against Gram-positive World Health Organization (WHO) priority pathogens such as VRE (*E. faecium*) and MRSA. Additionally, romsacin also eradicated the biofilms of VRE, MRSA, *Staphylococcus epidermidis*, and *S. haemolyticus*.

Nosocomial infections are mostly caused by MDR *E*. *coli*, enterococci, *P. aeruginosa*,* Acinetobacter baumannii*, *K. pneumoniae*, pneumococci, *S. aureus*, and *Proteus* spp. (Ghodhbane et al. 2015; Khan et al. 2017; Le et al. 2021; Rossi et al. 2024). Lacticin 3147, klebicin, and nisin A have shown high inhibitory activity against multiple nosocomial pathogens including MRSA and VRE (Piper et al. 2009; Ahmad et al. 2017; Alattar et al. 2024; Zhao et al. 2024). These bacteriocins also exhibit significant antagonism against pathogens in the kidney, liver, and spleen. In an in vivo study involving *S. aureus* Xen 29 infected mice, subcutaneous treatment with lacticin 3147 prevented the systemic spread of the pathogen, indicating the potential of lacticin 3147 as a biotherapeutic in real-life applications (Piper et al. 2009). Pumilicin 4, a bacteriocin produced by *Bacillus pumilus*, has shown remarkable inhibitory activity against MRSA, VRE, and several Gram-positive bacteria (Aunpad and Na-Bangchang 2007). This demonstrates the potential of the use of Pumilicin 4 in the management of infections caused by MRSA, VRE, and other susceptible Gram-positive pathogenic bacteria. Similarly, planosporicin, a bacteriocin produced by *Planomonospora* spp. DSM14920, has shown activity against *S. pyogenes*, *S. pneumoniae*, and *S. aureus* (Aunpad and Na-Bangchang 2007). Jabés et al. (2011) and Mota-Meira et al. (2005) separately demonstrated high in vitro and in vivo inhibitory activities of bacteriocins NAI-107, mutacin B-Ny266, and microbisporicin against MDR pathogens. Additionally, the activity of microcin J25, a bacteriocin produced by *E. coli* against multidrug-resistant Enterobacteriaceae has also been reported (Telhig et al. 2022).

The growth of major pathogenic bacteria including *H. influenzae*, *Pasteurella multocida*, *Mycobacterium tuberculosis*, *P. aeruginosa*, or *Moraxella catarrhalis*, responsible for various respiratory tract infections (RTIs) such as rhinitis, pneumonia, otitis, and tuberculosis were reportedly inhibited by different bacteriocins (mutacin B-Ny266, bacteriocin L23, lantibiotic MU1140, nisin F, and Mersacidin) under in vivo conditions in mice and Wistar Rats models and in vitro models (Kruszewska et al. 2004; Mota-Meira et al. 2005; Pascual et al. 2008; De Kwaadsteniet et al. 2009; Ghobrial et al. 2009; Le et al. 2023; Martin et al. 2023; Zhao et al. 2024). The activities of these bacteriocins under varied in vivo conditions, including immunosuppression, were observed to have no toxicity to the bronchi, trachea, lungs, or haematology of the evaluated animals. Similarly, purified salivaricin D and mutacin 1140 have shown antagonism against known RTI pathogens, *P. aeruginosa*, *S. aureus*, and *S. pneumoniae* (Ghobrial et al. 2009; Birri et al. 2012). Multiple in vitro and in vivo (mice and macrophages) anti-tubercular activities of various bacteriocins (e.g. lacticin 3147, nisin, laterosporulin10, and enterocin AS-48) have been tested against different strains of *M. tuberculosis* with favorable outcomes (Sosunov et al. 2007; de Kwaadsteniet et al. 2010; Carroll et al. 2010; Aguilar-Pérez et al. 2018)*.* Furthermore, variants of bioengineered nisin S, T, and V tested against *M. tuberculosis* (H37Ra), *M. avium* subsp. *Paratuberculosis* (ATCC 19698), *M. avium* subsp. *Hominissuis* (CIT05/03), and *M. kansasii* (CIT11/06) showed more significant inhibitory activities compared to parent nisin (Carroll et al. 2010). Among the bioengineered nisin variants, nisin S showed the most potent antagonism. Latham et al. (2017) also reported narrow-spectrum activity against nontypeable *Haemophilus influenzae* (NTHi) by a novel bacteriocin produced by *Haemophilus haemolyticus*. Their findings suggest that the novel bacteriocin or bacteriocinogenic strains of *H. haemolyticus* have the potential to reduce NTHi colonization and respiratory tract infection caused by NTHi.

Topical evaluation of bacteriocins has successfully been reported against oral and skin diseases, and breastfeeding women with mastitis (Fernández et al. 2008; Kang et al. 2009; Tong et al. 2014). Etiological agents of these diseases especially *Propionibacterium acnes*, *P. aeruginosa*, *S. aureus*, *S. epidermidis*, *L. monocytogene*s, *B. subtilis*, and *B. cereus* were controlled using bacteriocins such as nisin, lactocyclicin Q, subpeptin JM4B and hiracin JM79 (Sánchez et al. 2007; Kang et al. 2009; Sawa et al. 2009; Izquierdo et al. 2009; Ovchinnikov et al. 2020; Barman et al. 2023). Similarly, bacteriocins or bacteriocin-based formulas have been topically used for the treatment and prevention of mastitis and intramammary infections in animals Bennett et al. 2021; 2022; Heinzinger et al. 2023; Raheel et al. 2023). Several studies have reported the potency of different bacteriocins against pathogenic bacteria responsible for dental infections, vaginosis, gastric ulcers, gastroenteritis, etc. (Howell et al. 1993; Dover et al. 2007; Miyauchi et al. 2012; Kaewnopparat et al. 2013; van Staden et al. 2016; Cebrián et al. 2019; Ovchinnikov et al. 2020, 2021; Goodarzi et al. 2020; Benítez-Chao et al. 2021; Sharma et al. 2022; Barman et al. 2023; Alessandrini et al. 2023).

#### Potential antiviral agents

Apart from antibacterial properties exhibited by bacteriocins, several bacteriocins also possess antiviral activities against different viruses. While working with bacteriocins produced by *E. faecium* CRL35, (Wachsman et al. 1999) first described the antiviral activity of enterocin CRL35 against Herpes simplex viruses (HSV-1 and HSV-2). Enterocin CRL35 interferes with intracellular viral multiplication and inhibits viral late stages of replication (Wachsman et al. 2003; Al Kassaa et al. 2014). Similarly, enterocin ST4V and enterocin ST5Ha produced by *E. mundtii* ST4V and *E. faecium* ST5Ha, respectively, have shown high potency against HSV-1 and HSV-2 (Wachsman et al. 2003; Todorov et al. 2005). Bacteriocins produced by *L. curvatus* and *L. delbrueckii* subsp. *Bulgaricus* have shown antiviral properties against murine norovirus (MNV) and influenza virus (H1N1) (Serkedjieva et al. 2000; Lange-Starke et al. 2014). Non-LAB bacteriocins including Subtilosin A, erwiniocin NA4, and staphylococcin 188 produced by *B. subtilis*, *E. carotovora* NA4, and *S. aureus* AB188 independently showed inhibitory activities against HSV-1 (Torres et al. 2013), influenza, Newcastle disease, and coliphage HSA viruses (Qureshi et al. 2006; Saeed et al. 2007), respectively. Likewise, *Actinomadura namibiensis* DSM 6313 secretes bacteriocin, Labyrinthopeptin A1 (LabyA1) with antiviral activity against HSV and human immunodeficiency virus type 1 (HIV-1) (Férir et al. 2013). LabyA1 inhibited intracellular transmission of HIV-1 between infected and noninfected CD4 + T cells. Lee et al. (2016) similarly demonstrated the antiviral inhibitory activity of Micrococcin P1. In their study, they reported that Micrococcin P1, a naturally occurring macrocyclic peptide efficiently inhibited the attachment, entry, and cell-to-cell transmission of all hepatitis C virus (HCV) genotypes.

In a recent study, bacteriocin-like inhibitory substances produced by *E. faecium* CM019 isolated from Egyptian dairy products showed broad-spectrum antimicrobial activity against severe acute respiratory syndrome coronavirus 2 (SARS-CoV-2) and several Gram-positive bacteria activity (Bahy et al. 2023). Generally, the antiviral mechanisms and pharmacodynamics of bacteriocins against viruses are yet to be fully elucidated. However, it is believed that bacteriocins interfere with viral key determinants responsible for viral replication (Wachsman et al. 2003). Further studies are required to decipher the mechanisms of action and pharmacodynamics of bacteriocins against different viruses, especially those emerging with high virulence.

#### Potential non-invasive bio-diagnostic tool

Emerging reports show the great potential of bacteriocins as valuable tools for bioanalytical purposes in medicine, largely due to their precision, specificity, and in vivo recognition in biological systems. Different studies have demonstrated the labeling of bacteriocins using specific organic probes, fluorescent, or radioactive markers (Imran et al. 2013; Deng et al. 2020; Escobar et al. 2023). Through visualization with fluorescence ratio imaging microscopy, a labeled bacteriocin, fluorescent nisin Z, was able to precisely detect three pathogenic listerial strains: *L. monocytogenes* CIP 82110, *L. ivanovii* CIP 12510, and *L. innocua* CIP 12511 (Imran et al. 2013). Additionally, the mechanism of antilisterial action using the labeled nisin was demonstrated. Technetium-99 m (^99m^Tc)–duramycin a bacteriocin which is known to have high specificity and affinity towards phosphatidylethanolamine was used to identify apoptotic and necrotic cells (Ahmad et al. 2017). The combinatorial use of sodium iodide symporter (NIS) and ^99m^Tc-duramycin single-photon emission computed tomography (SPECT) imaging has proven effective in monitoring the spread of oncolytic virotherapy (OV) and determining the absence or presence of therapeutic-associated cell death (Zhang et al. 2019).

Recent advances in bacteriocin and peptide-based diagnosis, detection, and monitoring of pathogens have been increasingly developed for application in clinical and food systems with remarkable success. Various bacteriocins such as warnericin RK, leucocin, leucocin A, pediocin PA1, and curvacin A, have been used for the detection and monitoring of pathogens including bacteria and viruses, in clinical settings and the food system (Etayash et al. 2014a, b; Azmi et al. 2015; Islam et al. 2021, 2022; Escobar et al. 2023). These advances show the potential application of bacteriocins not only as noninvasive diagnostic tools for the diagnosis and prognosis of both infectious and non-infectious diseases but also for the identification of individuals predisposed to chronic diseases or secondary infections. Additionally, the use of peptide-based biosensors could offer promising, rapid, and highly sensitive alternatives for pathogen detection and food monitoring in agrifood systems.

#### Potential as anticancer agents

Globally, cancer remains one of the most severe, life-threatening, and difficult-to-treat diseases, resulting from the spread of uncontrollable proliferation of cells. The use of conventional cancer treatments, especially chemotherapy, radiotherapy, and surgery, often results in more devastating side effects and is still unable to curb the rising cases of cancer-associated morbidity and mortality (Naveen and Kalaivani 2018; Meade et al. 2020). A paradigm shift in cancer treatment approaches, including the use of innovative, safe, and sustainable solutions with no severe side effects is imperative. Interestingly, several bacteriocins have demonstrated varying degrees of anticancer activity (Hoskin and Ramamoorthy 2008; Kaur and Kaur 2015; Baindara et al. 2018; Meade et al. 2020). Due to the differences between the membranes of cancerous and healthy cells, bacteriocins can identify and selectively destroy cancer cells (Meade et al. 2020). Unlike healthy cells, which have outer membranes with neutral charged ions, the outer membrane of cancer cells upregulates the expressions of O-glycosylated mucins and phosphatidylserine (Yoon et al. 1996; Dobrzyńska et al. 2005) and becomes negatively charged. The negatively charged cell membranes of cancer cells trigger electrostatic interactions in the presence of [positively charged] bacteriocins (Hammami et al. 2010; Baindara et al. 2018; Meade et al. 2020; Ananou et al. 2020). The inhibitory activity of bacteriocins against cancer cells is primarily based on membrane permeabilization, which is mainly due to the amphiphilic and cationic nature of bacteriocins (Kaur and Kaur 2015; Perez et al. 2018). Ahmadi et al. (2017) reported antiproliferative activity of nisin against colon cancer SW480 cells. Nisin ZP induced anticancer activity, resulting in a high level of apoptosis in squamous cell carcinoma (HNSCC cells) with no histological damage, necrosis, fibrosis, or inflammation even after prolonged exposure to nisin ZP (Kamarajan et al. 2015). Similarly, nisin has shown activity in the control of oral cancer as well as in head and neck squamous cell carcinoma in in vivo mice studies (Lopetuso et al. 2019). Purified colicin, microcin, pediocin, and pyocin have also demonstrated high inhibitory activities in xenograft mouse models and neoplastic cell lines (Shin et al. 2016). Microcin E492, produced by *K. pneumoniae*, exhibits anticancer properties against breast and colorectal cancer cells through the induction of apoptosis and necrosis in some human cell lines (Hetz et al. 2002).

In recent years, several bacteriocins, including Laterosporulin10, Enterocin 12a, nisin A, Fermenticin HV6b, colicins, and Enterocin LNS18, have shown anticancer properties against various types of cancers in different cancer cell models (Baindara et al. 2017; Norouzi et al. 2018; Al-Madboly et al. 2020; Hosseini et al. 2020; Soleimanpour et al. 2020; Sharma et al. 2021; Balcik-Ercin and Sever 2022; Molujin et al. 2022; Ye et al. 2023). These bacteriocins often exhibit anticancer activities against human cell lines or in vivo, with minimal activity towards non-cancerous cells. Several studies have confirmed the anticancer potential of bacteriocins. However, more in vivo studies are necessary to fully elucidate and validate the clinical potency of bacteriocins as anticancer therapeutic agents.

### Food applications

The application of bacteriocins in the food system has been extensively studied since their discovery. Bacteriocins are naturally synthesized and ready-to-use, without color, taste, odor, or impact on the sensory properties of food. They also demonstrate stability at high temperatures and low pH, making them increasingly important in the food sector (Perez et al. 2014; Abbasiliasi et al. 2017; Yang et al. 2018; Sanguyo et al. 2021; Shafique et al. 2022; Field et al. 2023; Yu et al. 2023). The suitability of bacteriocins for extensive application in the food system leverages several beneficial aspects of food production. Bacteriocins are able to (a) decrease the risk of transmission of foodborne or zoonotic pathogens and food poisoning, (b) improve the shelf life of food, (c) decrease economic losses due to disease outbreaks, food spoilage, and recalls, (d) preserve the nutritional value of food through the reduction of the intensity of physical treatments, (e) decrease processing costs and time, (f) provide a safe and sustainable alternative preservation approach for ready-to-eat and "novel” food, and (g) provide extra protection during temperature abuse episodes (Gálvez et al. 2007; Hu et al. 2014; Darbandi et al. 2022). While various aspects of bacteriocin applications within the food system, including food preservation, fermentation, and protective culture, have been extensively reviewed (Deegan et al. 2006; Zacharof and Lovitt 2012; Perez et al. 2014; Bali et al. 2016; Ahmad et al. 2017; Lopetuso et al. 2019), we provide additional updates on the emerging and relevant potential of bacteriocin use in the food system.

#### Potential in antimicrobial food packaging

Despite the application of advanced technologies in the food industry, excessive economic loss as a result of microbial contamination and spoilage continue to constitute a major challenge globally. The application of antimicrobial agents, including bacteriocins, in antimicrobial packaging is specifically suitable for mitigating the risk of microbial contamination. The use of bacteriocin-coated packaging films to inhibit and control food spoilage has attracted considerable attention the recent years. These bacteriocins can either be directly coated onto the packaging film surface or incorporated into the matrix of the packaging film (Woraprayote et al. 2016; Ahmad et al. 2017; Benabbou et al. 2020). However, it is important to understand both the physicochemical properties and the mechanism(s) of action of the selected bacteriocin(s) for such use (O’Connor et al. 2015). Active bacteriocin coating serves to protect food products by continuously interacting with the packaged food and modifying the internal environmental conditions within the required shelf life (Gumienna and Górna 2021). In most instances, bacteriocins improve food quality by maintaining microbiological safety, improving nutritional and sensory properties, and extending shelf life (Santos et al. 2018; Mousavi Khaneghah et al. 2018; Sanguyo et al. 2021; Shafique et al. 2022; Yu et al. 2023). Food packaging films or polymers incorporated with bacteriocins directly inhibit the growth of microorganisms on the food surface, where most of the microbial food spoilage or contamination occurs (Ahmad et al. 2017; Gumienna and Górna 2021; Rivera-Hernández et al. 2021). Interestingly, most bacteriocins retain their antimicrobial activity during food processing. Their viability is not impacted by changes in temperature, sterilization, pasteurization, or other processing techniques (Santos et al. 2018; Gumienna and Górna 2021). The growing consumer demand for safe, natural, and chemical-free food has enabled food industries to explore the use of bacteriocins in food packaging, among other applications. Active bacteriocin-coated materials are highly promising sustainable solutions to enhance food safety and shelf life while retarding food contamination and spoilage.

For example, a polyethylene-based packaging film infused with plantaricin BM-1 produced by *L. plantarum* BM-1 showed antilisterial activity against *L. monocytogenes* for at least 120 days at room temperature (Zhang et al. 2017). Woraprayote et al. (2018) also demonstrated the inhibitory activity of *Weissella hellenica*-produced bacteriocin 7293 impregnated onto a biocomposite film (PLA/SP) with pangasius fish fillets against various foodborne pathogens, including *A. hydrophila*, *S. aureus*, *L. monocytogenes*, *P. aeruginosa*, and *S. typhimurium.* The adsorption of nisin on a wide variety of packaging films with antimicrobial activities has been successfully reported on polypropylene, ethylene vinyl acetate, polyethylene, polyvinyl chloride, acrylics, polyamide, and polyester. Nisin-incorporated coatings for poultry products have also been documented (Appendini and Hotchkiss 2002; Scaffaro et al. 2011; Tumbarski et al. 2018). Polyamide and polyethylene pouches coated with nisin preparation (Nisaplin^®^) and lacticin 3147 significantly reduced *L. lactis* subsp. *lactis*, *S. aureus*, and *L. innocua* during the storage of vacuum-packed cheese (Scannell et al. 2000). Pediocin coated on plastic bags and cellulose casings completely inhibited *L. monocytogenes* in meats during 3 months of storage at refrigeration temperature (Ming et al. 1997). Benabbou et al. (2020) also reported the antimicrobial properties of biocompatible and biodegradable chitosan films incorporated with divergicin M35 for the biocontrol of *Listeria* spp. in foods, especially minimally processed products, and ready-to-eat food. The success observed in these studies highlights the potential of bacteriocins in antimicrobial packaging by effectively inhibiting or limiting the growth of spoilage and pathogenic microorganisms in packaged food.

#### Potential as antibiofilm and sanitizers

Microorganisms mostly exist as sessile communities, known as biofilms, enclosed in an extracellular matrix typically composed of extracellular DNA, lipids, polysaccharides, etc. (Flemming et al. 2016). Biofilm formation by microorganisms in the food system makes them resistant to antimicrobials and difficult to remove from food production facilities, surfaces, and environments (Mathur et al. 2018). Many biofilm-forming species in the food industry are known human pathogens that can cause metal corrosion, changes in organoleptic properties of food, and disease (Colagiorgi et al. 2017; Kirtonia et al. 2021). Biofilms are commonly found on surfaces such as tanks, pipelines, glass, polyethylene, polypropylene, rubber, packaging tools, and wood (Kirtonia et al. 2021). Recently, the use of bacteriocins as antibiofilm agents in the food industry has been widely reported (Mathur et al. 2018; Kirtonia et al. 2021; Jiang et al. 2022; Zhang et al. 2022a, b). In a study by Bolocan et al. (2017), several bacteriocins including, subtilomycin, nisin Z, and lichenicidin demonstrated high antibiofilm activity against *L. monocytogenes* biofilms. These bacteriocins also significantly decreased the viability of already formed biofilms. Another study showed that nisin at the concentration of 4000 IU/ml reduced biofilm formation by 87, 57, and 30% for *Salmonella* Enteriditis, *L. monocytogenes*, and *S. aureus*, respectively (Mahdavi et al. 2007). Bacteriocin sonorensin exhibited inhibitory activity against *S. aureus* biofilms (Chopra et al. 2015). From their study, the inhibitory property of sonorensin was attributed to increased membrane permeability in *S. aureus*. Biofilms formed by fourteen *Staphylococcus* strains were inhibited by hyicin 4244, a circular sactibiotic secreted by *S. hyicus* 4244 (Duarte et al. 2018). Hyicin 4244 decreased biofilm-forming ability, number of cells, cellular viability, and proliferation of sessile cells within already formed biofilm.

While the combination of nisin with enterocin B3A-B3B resulted in a 2-log decrease in *L. monocytogenes* biofilms on the surface of stainless steel within 24 h, nisin mixed with ethanol however resulted in a 5-log reduction of *Salmonella* and *E. coli* biofilms on stainless steel surfaces within 15 min (Phongphakdee and Nitisinprasert 2015; Al-Seraih et al. 2017). Industrial application of bacteriocins as antibiofilm agents or sanitizers may require a longer period to achieve significant bacterial reduction. However, bacteriocin combination with other antimicrobials can result in rapid bacterial reduction and biofilms clearance. Further studies are needed to explore the potential of bacteriocins as antibiofilm agents in the food industry, focusing on unraveling their mechanism of action and spectrum of activity.

### Agriculture and veterinary medicine

Antibiotics have been routinely used in agriculture, either for treating or preventing animal diseases or as growth promoters. This practice has significantly contributed to the increased emergence and spread of antimicrobial-resistant pathogens from animals to humans (Ben Lagha et al. 2017). To address the issue of AMR in animal production, many countries have prohibited antibiotic use as growth promoters in animal production (European Commission 2005; AccessScience Editors 2017; Prescott 2019; Field et al. 2023; WOAH 2023). Therefore, the application of bacteriocins and/or bacteriocin-producing strains as growth promoters, prophylaxis, or therapeutics in agriculture has been considered viable and sustainable alternatives to antibiotics.

#### Potential as prophylactic and therapeutic agents

Dairy animals often suffer from mastitis, which is an inflammation of the mammary gland resulting in considerable economic losses due to reduced milk quantity and quality. Mastitis is predominantly caused by *S. aureus*, *S. dysgalactiae*, *S. uberis*, *Mycoplasma* spp., and *E. coli* (Cheng and Han 2020). Several bacteriocins, including lacticin 3147 and nisin, have been shown to inhibit the etiological agents of mastitis, especially *S. agalactiae* and *S. aureus* in dairy cattle (Cao et al. 2007; Pieterse et al. 2010b; Klostermann et al. 2010; Field et al. 2021; Bennett et al. 2021; 2022; Heinzinger et al. 2023; Raheel et al. 2023). The United States FDA has approved the general use of a nisin-based preparation, Wipe Out^®^ Dairy Wipes (Immucell, Portland, ME, USA), for mastitis control in lactating dairy cows. Klostermann et al. (2010) demonstrated the efficacy of lacticin 3147 in eliminating mastitis-causing *S. uberis*, *S. dysgalactiae*, and *S. aureus* after a 10-min teat dip treatment. Other bacteriocins, such as aureocins A70, A53, epilancin K7, entomocin, Pep5, kurstacin 287, bacteriocin ST91KM, uberolysin, nisin U, kenyacin 404, and epidermin, have shown anti-mastitis effects against *S. aureus* and *S. agalactiae* (Barboza-Corona et al. 2009; Pieterse et al. 2010a; Salvucci et al. 2012).

Microcin J25 has been used for *Salmonella* control in poultry (Stavric and D’Aoust 1993; Ben Said et al. 2020; Baquero et al. 2024). Divercin AS7, a bacteriocin produced by *Carnobacterium divergens* AS7 has been effective in controlling *S. enterica* Typhimurium, *Campylobacter* spp., and *C. perfringens* in both poultry and swine (Gillor et al. 2004; Stern et al. 2005; Udompijitkul et al. 2012). Our recent studies have demonstrated the antagonistic and pathogen-reducing activity of plantaricin EF producing-*L. plantarum*, alone and in combination with other potential probiotic strains against enterobacteria in poultry (Reuben et al. 2022) as well as other zoonotic pathogens such as *Salmonella* Typhimurium, *S.* Enteritidis, *E. coli* O157: H7, *E. faecalis*, and *L. monocytogenes* (Reuben et al. 2020). In another study involving boilers challenged with *Pasteurella multocida*, we found that dietary supplementation with novel multistrain probiotics containing plantaricin EF-producing *L. plantarum* attenuated mortality, clinical manifestations, and inflammatory reactions associated with *P. multocida*-induced fowl cholera (Reuben et al. 2021). Furthermore, the abundance of gut enterobacteria and *P. multocida* was also significantly reduced in birds supplemented with the multistrain probiotics containing plantaricin EF-producing *L. plantarum.* Similarly, the therapeutic potential of bacteriocin and a strain of bacteriocin producing *L. plantarum* was investigated on broilers experimentally infected with *E. coli* (Ogunbanwo et al. 2004). Treatment with bacteriocin or the producing *L. plantarum* strain reduced *E. coli*-associated infections and improved the overall health and well-being of the birds.

#### Potential as growth promoters

The prohibition of antibiotic use as growth promoters in animal production has created a void that must be filled with equally potent, safe, and sustainable alternatives. Bacteriocins and their producing strains have emerged as widely accepted and suitable growth promoters in animal production. Several studies have demonstrated the growth promotion effects of bacteriocins and bacteriocin-producing strains in various animal species including poultry, cattle, and swine (Gillor et al. 2004; Cutler et al. 2007; McAllister et al. 2011; Józefiak et al. 2013; Reuben et al. 2021, 2022; Soltani et al. 2022a; Zhang et al. 2022a, b; Field et al. 2023).

The dietary supplementation with colicin E1 improved growth performance and significantly reduced F18-positive enterotoxigenic *E. coli*-associated postweaning diarrhea in piglets (Cutler et al. 2007). Supplementation with *L. salivarius* Bacteriocin Abp118 induced intestinal microbiota modulation, leading to increased growth performance and feed conversion efficiency in pigs (Riboulet-Bisson et al. 2012). Grilli et al. (2009) observed improved growth performance in *C. perfringens* infected broiler chickens supplemented with pediocin A alone or in combination with the producing strain. Similarly, the inclusion of nisin in the diet of broiler chickens beneficially modulated gut microbiota and significantly enhanced feed conversion and growth performance (Józefiak et al. 2013). Supplementation with plantaricin EF-producing *L. plantarum*, alone or in combination with other probiotic strains including *E. faecium* C14 and *P. pentosaceus* I13, improved haemato‐biochemical parameters, intestinal health, and growth in broilers (Reuben et al. 2022). Dietary supplementation of broiler feed with bacteriocin microcin J25 significantly improved performance, intestinal microbiota composition, and diversity, while reducing systemic inflammatory markers and levels of faecal *E. coli* and *Salmonella* (Wang et al. 2020b). These studies demonstrate the potential of bacteriocins or bacteriocinogenic strains as viable alternatives to antibiotics for growth promotion in animals.

#### Potential in sustainable aquaculture

The aquaculture supply chain is continuously exposed to multiple physical, chemical, and biological hazards, especially a wide range of pathogenic organisms. This impacts the quality and safety of aquaculture and its products. Minimizing microbiological hazards often involves the use of antibiotics, which enhances the selective pressure for the emergence and spread of superbugs and drug residues in both aquaculture products and their environment (Gillor et al. 2008; Wang et al. 2019a; Stentiford et al. 2022). However, in recent years, substantial attention has been given to the use of bacteriocins in aquaculture mostly for aquaculture processing and disease mitigation, improvement of water quality, and enhancement of sensory quality and shelf life (Wang et al. 2019a). Bacteriocin cloning and heterogeneous expressions from producing strains have demonstrated great potential in designing robust microbial cell factories capable of producing potent bacteriocins (Xu et al. 2019; Feito et al. 2023). Through this advancement, Feito et al. (2022) and Contente et al. (2023) engineered a recombinant multi-bacteriocinogenic strain (*L. cremoris* WA2-67) to produce three bacteriocins: garvicin A, Q, and nisin Z. The three recombinant bacteriocins, especially nisin Z, beneficially enhanced immune functions and growth performance while inhibiting pathogen colonization in rainbow trout (*Oncorhynchus mykiss*, Walbaum) (Contente et al. 2023). Bacteriocin-like substances (BLS) obtained by co-cultures of *E. faecium* MU8 with *Aeromonas veronii* showed significant antimicrobial activity against major pathogens of *Nile tilapia*, including *Aeromonas jandaei* and *A. veronii* (Promrug et al. 2023). Bacteriocin production through co-cultures of Gram-negative-inducing strains with Gram-positive bacteriocin-producing strains is now used to increase bacteriocin biosynthesis and yields (Liu et al. 2021; Promrug et al. 2023).

Bacteriocins such as enteromycin F4-9 and MC13, produced from *E. faecalis* F4-9 and *E. faecium* MC13 respectively, have shown broad inhibitory activity against both Gram-negative and Gram-positive bacterial pathogens of aquatic animals, including *E. coli* JM109, *A. hydrophila*, *Vibrio harveyi*, and *V. parahaemolyticus* (Pinto et al. 2009). Bacteriocin produced by *A. media* strain A199 has controlled *V. tubiashii*-infected Pacific oyster larvae (Gibson et al. 1998) and significantly reduced mortality due to saprolegniosis in eels (Lategan and Gibson 2003). The dietary inclusion of bacteriocin NPUST1 produced by *Paenibacillus ehimensis* NPUST1 reduced the counts of *S. iniae* and *A. hydrophila* and improved the growth performance of *Oreochromis niloticus* (Nile tilapia) (Chen et al. 2019). Plantaricin FGC-12 applied to Whiteleg shrimp (*Penaeus vannamei*) inhibited *V. parahaemolyticus* by causing cell wall perforation (Hu et al. 2013).

Furthermore, bacteriocin-like substances obtained from LAB associated with the gut of *Mugil cephalus* L (grey mullet) improved water quality, inhibited the growth of *L. garvieae* and reduced microbial-associated morbidity and mortality in aquatic animals (Lin et al. 2013). In addition to their pathogen inhibitory properties, bacteriocins also improve the sensory properties and shelf life of aquatic products (Cortesi et al. 2009; Alzamora et al. 2012).

#### Potential as plant growth promoters

So far, only bacteriocins of *Bacillus* spp. have been extensively studied and mostly used in plant production (Nazari and Smith 2020; Negash and Tsehai 2020). Bacteriocins bacthuricin F4 and thuricin 17 are produced by different *B. thuringiensis* strains, especially *B. thuringiensis* BF4 and NEB17. These bacteriocins, along with bacteriocin C85 secreted by *B. cereus* UW85, have been reported to possess growth promotion properties in plants (Negash and Tsehai 2020). Applying a cocktail containing the combination of the 3 bacteriocins and their producing strains increased photosynthesis by 6%, plant dry weight by 15%, root nodulation by 21%, and leaf area in corn, soybean, and tomato plants when compared with controls. These bacteriocins exhibit bacteriocidal and bacteriostatic activities that promote disease resistance in plants.

Mirzaee et al. (2021) recently reported that plant-produced bacteriocins inhibit different plant pathogens while conferring resistance to diseases in tomatoes. Furthermore, other bacteriocins such as amylocyclicin, Bac 14B, Bac-GM17, putidacin, and cerein 8A have been used for both antimicrobial activity and growth promotion in plants (Cherif et al. 2001, 2008; Hammami et al. 2009; Prudent et al. 2015).

## Commercialization of bacteriocins: patent and market perspectives

**Fig. 4:**
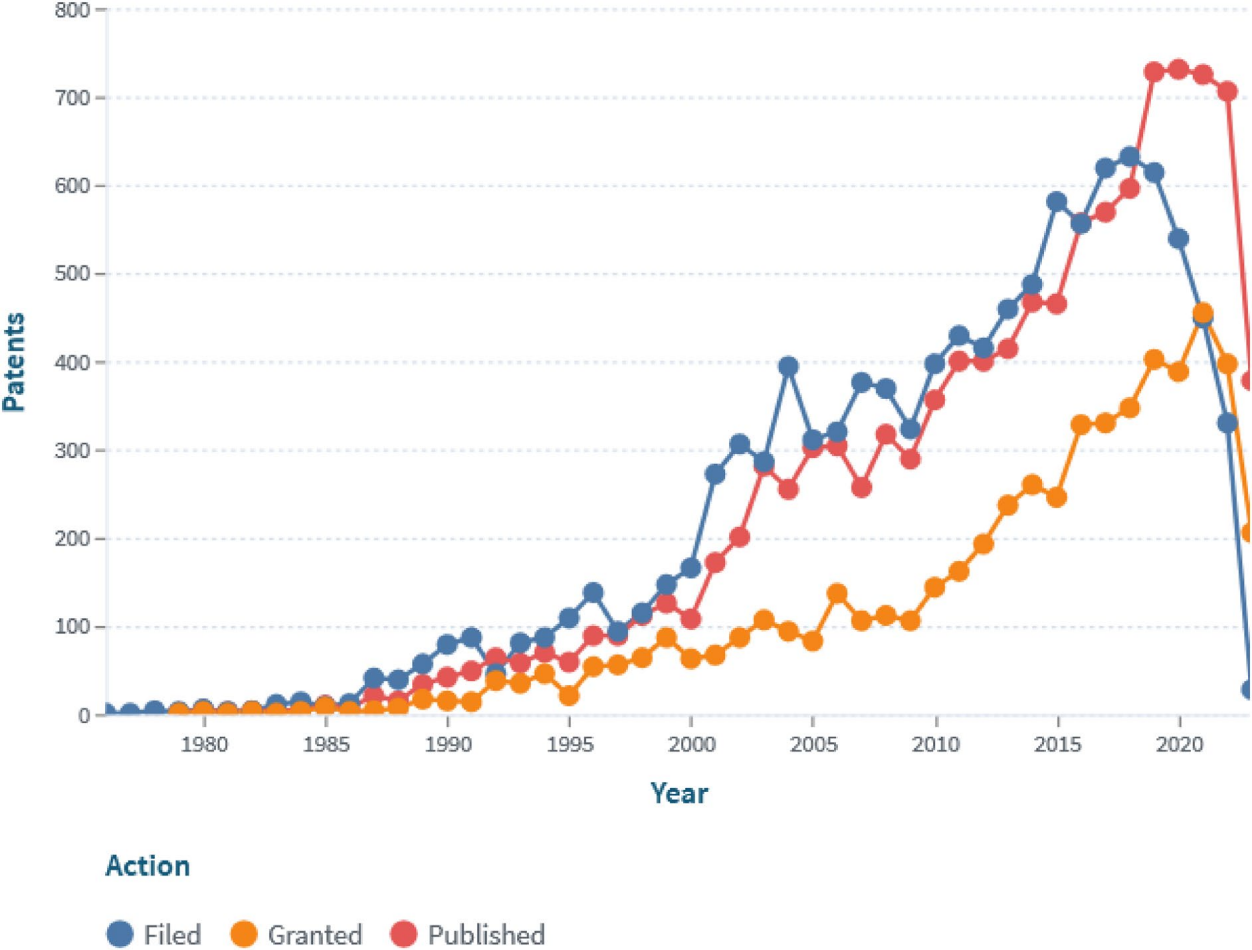
Bacteriocin patent publication, filing, and approval

While the current report of the World Intellectual Property Organization (WIPO) (https://www.wipo.int/portal/en/index.html) shows 1127 bacteriocins-related patent applications published, the Espacenet and Lens global patent search engines (https://www.epo.org/ and https://about.lens.org/) report 10,790 and 10,846 patents, respectively (Fig. 4). Over the past three decades, there has been a consistent increase in bacteriocin-related patent publications, filings, and approvals. The leading countries in patent applications are the USA, China, Canada, the Republic of Korea, Japan, and Australia. The top applicants include Colgate Palmolive Co, Unilever Plc, Unilever Nv, University of California, Coca-Cola Co, Chr Hansen As, and US Agriculture (Figures S2 and S3). The fascinating properties of bacteriocins contribute to their widespread acceptance and market potential.

In 1969, the Food and Agriculture Organization/World Health Organization (FAO/WHO) of the United Nations approved the general use of nisin as a food preservative. Subsequently, the European Union (Directive 83/463/EEC; Directive 95/2/EC), United States (FDA 21CFR), and Canada [Health Canada (NOP/ADP-0028)] granted similar approvals in 1983, 1988, and 2017, respectively. Although most commercially available bacteriocins especially nisin (Nisaplin™, Biosafe™, Oralpeace™), leucocin A (Bactoferm™ B-SF-43), sakacin (Bactoferm™ B-2, BactofermTM B-FM), and pediocin PA-1 (Microgard™, Alta 2341), are primarily used as food preservatives (Daba and Elkhateeb 2020; Cesa-Luna et al. 2021; Naskar and Kim 2021), others such as colicins and salmocins, intended for use as antibacterial agents, have received FDA approval (Hahn-Löbmann et al. 2019). In 2019, the FDA approved five bacteriocin preparations including, SalE1a, SalE1b, SalE2, SalE3, and SalE7 (Nomad Bioscience GmbH) for use as antimicrobial agents on meat, poultry, fish, and egg products (https://www.fda.gov/media/135524/download) The five bacteriocins were derived from non-typhoidal bacteriocinogenic *Salmonella* strains, and can be used individually or in combination. Duramycin (Moli1901) (AOP Orphan Pharmaceuticals AG), a commercially available bacteriocin, has been used in patients for the treatment of cystic fibrosis in humans (Grasemann et al. 2007; Steiner et al. 2008). Similarly, Delvo^®^ Nis (DSM, Delft, Netherlands), Nisin Z^®^ (Handary, Brussel, Belgium), and Nisaplin^®^ (Danisco, Copenhagen, Denmark) have been approved for commercial use (Soltani et al. 2021a).

Bacteriocin-producing protective cultures, such as *C. divergens* M35, *Leuconostoc carnosum* 4010 (Danisco, HOLDBAC^®^) and *C. maltaromaticum* CB1 have been approved by several countries for use in the food industry (https://members.wto.org/crnattachments/2017/SPS/CAN/17_0131_00_e.pdf; https://www.canada.ca/en/health-canada/services/food-nutrition/public-involvement-partnerships/use-microbiological-preparation-carnobacterium-maltaromaticum-strain-certain-ready-meat-poultry-products/document.html). Nisin-based commercially available bacteriocins approved by the USDA, Teatseal^®^ (Zoetis, USA), Wipe-Out^®^ Dairy Wipes and Mast Out^®^ (Immucell Corporation, USA) are commonly used as anti-mastitis agents in dairy cows (Soltani et al. 2021a). Additionally, nisin-incorporated soy-derived packaging films have been commercialized as an antimicrobial food package to inhibit *Listeria* (Ahmad et al. 2017). Several bacteriocin products, including sakacin (Bactoferm FLC^®^, Chr. Hansen, Hørsholm, Denmark), NVB302, Moli1901 (*Actinoplanes liguriae* NCIMB41362), mutacin 1140 (*S. mutans* JH1000), pediocin (Fargo 23, Quest International, B.V.), and NAI-107 (*Microbispora corallina*), are currently at various phases of clinical trials for subsequent use in health and agrifood systems (Soltani et al. 2021a; Cesa-Luna et al. 2021).

## Challenges and limitations of bacteriocin application and future research

Despite increasing research on bacteriocin discovery, characterization, and application over the past decades, only a few have been commercially applied. While bacteriocins are generally believed to be safe, concerns about their cytotoxicity against eukaryotic cells, stability, immunogenicity, development of resistance, unpredictable biofunctions, and high production costs have raised doubts about their application. These concerns necessitate extensive safety evaluations of each bacteriocin before final approval and use in health and agrifood systems.

Several reports have demonstrated the safety and non-cytotoxicity of bacteriocins; however, others have shown varying (low) degrees of cytotoxicity in both in vitro and in vivo experiments (Pulse et al. 2019; Baños et al. 2019; Cebrián et al. 2019, 2023; Wang et al. 2022; Abdille et al. 2022; Heinzinger et al. 2023). The minimal cytotoxicity observed in most studies was due to significantly higher concentrations of bacteriocins and prolonged experimental exposure, beyond the required minimum inhibitory concentrations (MIC) for pathogen inhibition or food protection. At significantly higher concentrations (above the MIC), bacteriocin PA166 showed minimal cytotoxicity on Vero and NR8383 cells, as well as in the mouse infection model (Wang et al. 2022). Similarly, enterocin AS-48, bacteriocin OG716, and dermaseptin only exhibited mild cytotoxicity with prolonged treatment or at significantly higher concentrations in Golden Syrian hamsters, B2 BALB/c mice, and Albino Wistar rats (Pulse et al. 2019; Baños et al. 2019; Abdille et al. 2022). Additionally, cytolysin, a bacteriocin produced by *E. faecalis*, showed broad cytotoxicity to various cell lines, including intestinal epithelial cells, leucocytes, erythrocytes, and human retinal cells (Coburn and Gilmore 2003; Cox et al. 2005*).* It is important to note that the cytotoxicity of bacteriocins can be influenced by factors such as purity, concentration, the specific mammalian cell line or experimental model used, and host-associated factors (e.g., in vivo experiments) (Cavicchioli et al. 2018; Soltani et al. 2021a; Cebrián et al. 2023). Certain eukaryotic cell lines may be more sensitive to particular bacteriocins than others, with differences attributed to cell type, composition of cell membranes, permeability, and hydrophobicity (Das and Goyal 2014; Soltani et al. 2021a; Abdille et al. 2022).

Physiological and physicochemical parameters can influence the stability and bioactivity of bacteriocins in the host or food matrix. Several reports have shown rapid inactivation or enzymatic degradation of bacteriocins produced in situ, orally ingested, or applied to food matrices (De Vuyst and Leroy 2007; Fernandez et al. 2013; Md Sidek et al. 2018; Holcapkova et al. 2018; Flynn et al. 2019, 2022; Soltani et al. 2021b). Despite their potential for applications in clinical and agrifood systems, class II bacteriocins are highly sensitive to proteolytic enzymes, which reduces their bioactivity when used (Soltani et al. 2021a). For instance, pediocin PA-1, nisin A, and microcin J25 were inactivated or degraded when exposed to intestinal contents and proteolytic enzymes (Kheadr et al. 2010; Gough et al. 2017; Naimi et al. 2018). However, through encapsulation and bioengineering, microcin J25 or nisin showed some stability in the presence of proteolytic enzymes and under intestinal conditions (Field et al. 2015, 2019). Engineering bacteriocins can help create resistant bacteriocin derivatives that can withstand harsh gut conditions and enzymatic degradation while maintaining their bioactivity. Additionally, systems like encapsulation and coating have been developed to protect and precisely deliver bacteriocins to the intended site of action or within specific food matrices, allowing them to exert their biological functions (Gomaa et al. 2017; Gough et al. 2018; Holcapkova et al. 2018; Flynn et al. 2019, 2022).

To avoid any sudden or unexpected immune responses, the immunogenicity of bacteriocins should be carefully examined, especially when intended for use in humans and animals. Generally, several bacteriocins, including pyocins S2, S5, AP41, and L1, bacteriocins LR14, TSU4, JCM1132, and P34, plantaricin E/F, mutacin 1140, microbisporicin, actagardine, and duramycin have been reported to be non-immunogenic in in vivo studies (McCaughey et al. 2016; Ongey et al. 2017; Sahoo et al. 2017; Hanny et al. 2019; Wang et al. 2019c). However, prolonged administration of some bacteriocins, such as pyocin S5 and Nisaplin^®^, has been shown to elicit mild immunogenicity (de Pablo et al. 1999; Scholl and Martin 2008; McCaughey et al. 2016). Furthermore, some bacteriocins have also displayed unique and unpredictable properties, expressing both bacteriocin and virulence factors. Listeriolysin S (LLS) and pneumocins exhibit both virulence and bacteriocin properties and are highly expressed in the gut of orally infected mice (Quereda et al. 2016; Wholey et al. 2019). Both LLS and pneumocins are antibiotic-induced and can alter the host intestinal microbiome, enhancing intestinal colonization with *L. monocytogenes* and *S. pneumoniae* (Kjos et al. 2016; Quereda et al. 2016; Wholey et al. 2019). Bacteriocins may exert different sudden and unpredictable effects when used in humans and animals. Therefore, their immunogenicity and other emerging co-bioactive properties such as virulence factors should be elucidated before use.

Another major challenge of bacteriocin application in health and agrifood systems is their low yields and high cost of large-scale industrial production, purification, and prolonged storage. For commercial and economic purposes, bacteriocins need to be produced in large and sufficient quantities. For research purposes, crude, unpurified, and concentrated bacteriocins are often produced using costly and complex media that are mostly not food or pharmaceutical-grade (Garsa et al. 2014; Abbasiliasi et al. 2017; Johnson et al. 2018). The bottleneck for efficient and commercial production of bacteriocins is the need for complex media that optimally support the metabolism and auxotrophies of the producing strains (Ongey and Neubauer 2016; Goldbeck et al. 2021). Additionally, industrial-level purification and biopreservation of bacteriocins is another limitation for commercial-scale bacteriocin production. In most cases, laboratory-based purification protocols are usually not suitable at the industrial scale mostly due to the high cost of the purification processes (Garsa et al. 2014; Mesa-Pereira et al. 2018; Juturu and Wu 2018). Nevertheless, chemical synthesis has been recently proposed as a viable alternative for the industrial-scale production of bacteriocins (Bédard and Biron 2018; Bédard et al. 2018; Desiderato et al. 2023; Sevim and Güneş Altuntaş 2024). Industrial and large-scale production of active bacteriocins using chemical synthesis would further enhance the use of bioengineering and consequently, improve stability, spectra of antimicrobial activity, and pharmacological properties of bacteriocins in humans and agrifood systems (Bédard et al. 2018; Kuniyoshi et al. 2022; García-Vela et al. 2024). Efficient and cost-effective production and purification processes are essential for wider applications in health and agrifood systems. Additional research is necessary to further develop economical and low-cost production and purification processes of highly promising bacteriocins.

Finally, like other conventional antimicrobials, persistent exposure to bacteriocins can lead to the development of resistance in target bacteria. Bacteriocin resistance has been demonstrated for divercin V41, mesenterocin, leucocin A, pediocin and pediocin-like bacteriocins, lacticin 3147, lysostaphin, nisin, pyocin S2, mesenterocin, mundticin KS, etc. (Sakayori et al. 2003; Opsata et al. 2010; Collins et al. 2012; Inglis et al. 2016; López-González et al. 2018; Bhattacharya et al. 2019; Gradisteanu Pircalabioru et al. 2021). So far, bacteriocin resistance has been mostly studied in in vitro and model systems and can either be acquired (emerged from previously susceptible strains) or innate (naturally inherent in taxonomically related strains) (Bastos et al. 2015; Soltani et al. 2021a). Bacteriocin resistance mechanisms can include impermeability due to changes in cellular surfaces, enzymatic inactivation, changes in the antimicrobial peptide targets, entrapment by secreted molecules that can bind and neutralize bacteriocins, chemical modifications in membrane lipid composition, D-alanylation of teichoic acid, cellular filamentation, efflux pumps, and capsule synthesis to avoid contact with bacteriocins (Sakayori et al. 2003; Chifiriuc et al. 2014; Bastos et al. 2015; Kumariya et al. 2015, 2019; Soltani et al. 2021a). Rasch and Knøchel (1998) and Collins et al. (2010) separately reported up to 5.2 and 8.0% resistance of multi-sourced *L. monocytogenes* to pediocin PA-1 and pediocin-like bacteriocins respectively. The instances and mechanisms of bacteriocin resistance have been extensively reviewed (Bastos et al. 2015; Gradisteanu Pircalabioru et al. 2021; Soltani et al. 2021a). Some target microbial strains have developed multiple mechanisms of resistance which can be synchronously displayed against specific bacteriocins (Vadyvaloo et al. 2002; Lohans and Vederas 2012; Bastos et al. 2015; Kumariya et al. 2015). This can therefore lead to the emergence of bacteriocin-resistant phenotypes, which may constitute an additional burden to the rising antimicrobial resistance menace. Understanding these resistance mechanisms and developing countermeasures can significantly enhance the clinical application of bacteriocins. Additional research is needed to address these challenges and further develop safe, economical, and low-cost production and purification processes for bacteriocins.

## Conclusion and prospects

The exacerbating global crisis of the emergence and spread of pathogens, antimicrobial resistance, dearth of novel antimicrobials, and the implementation of strict antibiotic-limiting policies in many countries necessitate a comprehensive approach to identify and apply widely accepted, potent, and safe alternative antimicrobials. Bacteriocins have invaluable and heterogeneous properties that make them suitable for use in human, animal, and food systems for disease prevention and treatment, microbiome modulation, growth promotion, and enhancing food quality, safety, and organoleptic properties, among other benefits.

Through bibliometric analyses, we have identified several prevailing trends in bacteriocin research. Firstly, there has been a significant increase in annual research outputs, which we believe reflects the growing global interest in bacteriocins. Secondly, we have observed a multidisciplinary participation in bacteriocin research, with contributions from various fields. Additionally, we have found that funding for bacteriocin research is relatively evenly distributed worldwide. The countries leading in bacteriocin-related research outputs are spread across the Northern and Southern Hemispheres. The majority of these research outputs are published by reputable publishes such as Elsevier, Springer Nature, Wiley, and the American Society of Microbiology. Interestingly, while bacteriocins research is primarily focused on microbiology, biotechnology, and food science, we have also discovered a significant number of outputs in emerging areas such as plant science, virology, polymer science, and biophysics. This suggests that bacteriocins may have applications in previously unknown fields, and we anticipate further research and applications in these areas in the coming years.

Harnessing the ubiquitous nature of bacteriocins could help in their exploitation for broad applications in innovative areas of the human, animal, and food systems. The food system is benefiting immensely from commercially available bacteriocins. However, the scope of bacteriocin applications in the food system could be expanded in areas such as [fermented] food microbiota modulation, antimicrobial packaging/coating, biosanitizers, antibiofilm, pre/post-harvest biocontrol, and functional food.

Furthermore, the use of bacteriocins in the modulation of human and animal microbiota can beneficially improve the composition, diversity, and richness of the microbiota, fostering health and well-being. Bacteriocin modulatory activity can provide a viable microbiome-based solution for the treatment and management of microbiome-associated diseases. Bacteriocins also have the potential for non-invasive bio-diagnosis and could be used for diagnosing both infectious and non-infectious diseases, thus complementing conventional diagnostic tools. In terms of agriculture, the growth-promoting effect of bacteriocins in both plants and animals would undoubtedly improve food security, safety, and quality, as well as promote sustainable agriculture and mitigate concerns associated with antibiotic use.

Through vigorous research, it is necessary to increase the potency and applications of bacteriocins in humans, using innovative approaches such as bioengineering, computational methods, artificial intelligence, nanotechnology, machine learning, microscopy techniques, chemistry, metabolic activity-based assays, and pharmacodynamics. These approaches will facilitate optimal and industrial-scale production of safe bacteriocins for general use.

### Supplementary Information

Below is the link to the electronic supplementary material.

Supplementary file1 (DOCX 312 KB)

## Data Availability

All data supporting the findings of this study are available within the paper.
