# Supplementary material for: Bacteriocins: potentials and prospects in health and agrifood systems
Source: Arch Microbiol. 2024 Apr 25;206(5):233. doi: 10.1007/s00203-024-03948-y (PMC11045635; doi:10.1007/s00203-024-03948-y)
Supplement: Supplementary file 1 — Supplementary file1 (DOCX 312 KB) [file 203_2024_3948_MOESM1_ESM.docx]

**Supplementary materials**


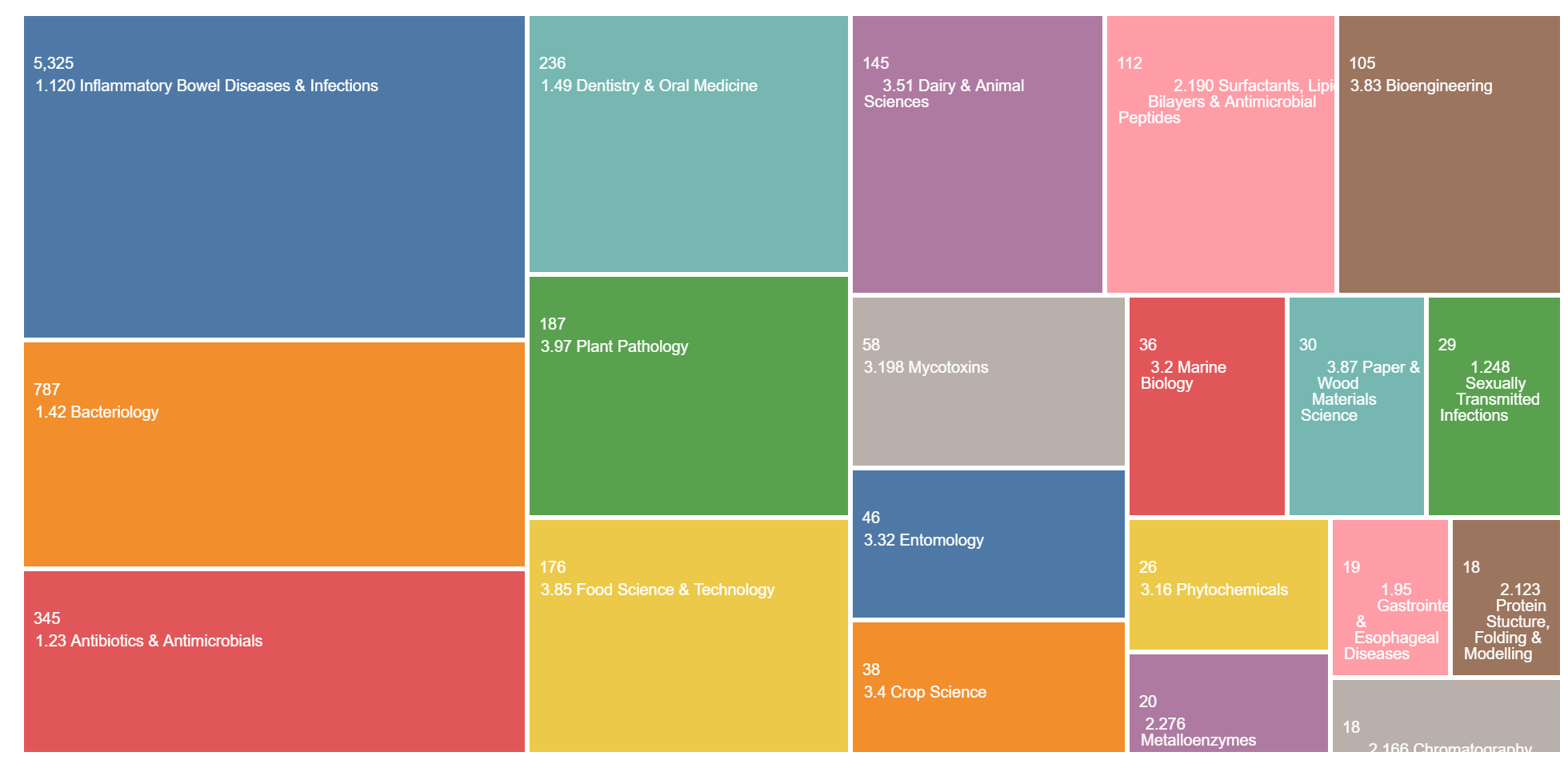


**Figure S1:** TreeMap representation of citations of bacteriocin-related publications across specialized areas


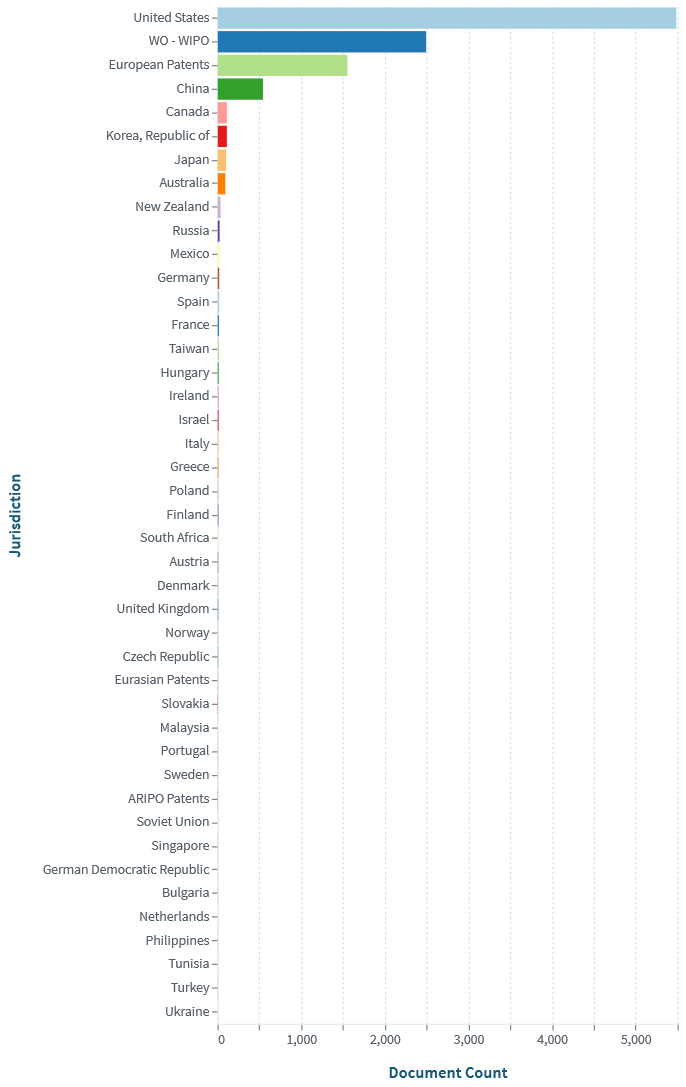


**Figure S2:** Bacteriocin patent application country/region


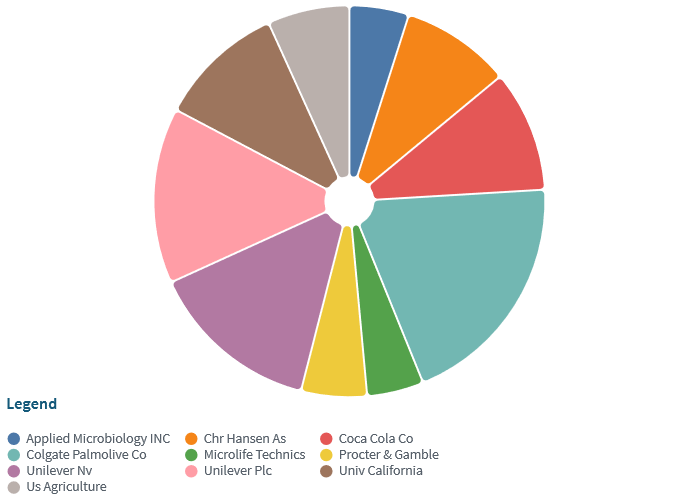


**Figure S3:** Bacteriocin patent top applicant

**Table S1:** Languages of bacteriocin-related published articles included in the synthesis

| # | Languages | Record Count | % of 8,303 |
| --- | --- | --- | --- |
| 1 | English | 8187 | 98.378 |
| 2 | French | 25 | 0.3 |
| 3 | Spanish | 22 | 0.264 |
| 4 | German | 21 | 0.252 |
| 5 | Russian | 18 | 0.216 |
| 6 | Czech | 9 | 0.108 |
| 7 | Japanese | 6 | 0.072 |
| 8 | Korean | 6 | 0.072 |
| 9 | Portuguese | 6 | 0.072 |
| 10 | Polish | 5 | 0.06 |
| 11 | Turkish | 5 | 0.06 |
| 12 | Chinese | 4 | 0.048 |
| 13 | Italian | 4 | 0.048 |
| 14 | Slovak | 2 | 0.024 |
| 15 | Hungarian | 1 | 0.012 |
| 16 | Welsh | 1 | 0.012 |

**Table S2:** Top 50 publishers of bacteriocins research

| Publishers | Record Count | % of 8,303 |
| --- | --- | --- |
| Elsevier | 1723 | 20.747 |
| Springer Nature | 1202 | 14.473 |
| Wiley | 914 | 11.005 |
| Amer Soc Microbiology | 905 | 10.897 |
| MDPI | 323 | 3.889 |
| Frontiers Media Sa | 240 | 2.89 |
| Taylor & Francis | 200 | 2.408 |
| Oxford Univ Press | 158 | 1.902 |
| Amer Chemical Soc | 106 | 1.276 |
| Microbiology Soc | 106 | 1.276 |
| Int Assoc Food Protection | 90 | 1.084 |
| Public Library Science | 87 | 1.048 |
| SOC GENERAL MICROBIOLOGY | 60 | 0.722 |
| Korean Soc Microbiology & Biotechnology | 57 | 0.686 |
| Academic Journals | 46 | 0.554 |
| NATURE PORTFOLIO | 40 | 0.482 |
| Amer Phytopathological Soc | 38 | 0.458 |
| Dr M N Khan | 38 | 0.458 |
| INT ASSOC MILK FOOD ENVIRONMENTAL SANITARIANS, INC | 38 | 0.458 |
| Bentham Science Publ Ltd | 36 | 0.433 |
| Cambridge Univ Press | 35 | 0.421 |
| AMER ASSOC DENTAL RESEARCH | 34 | 0.409 |
| Amer Soc Biochemistry Molecular Biology Inc | 34 | 0.409 |
| Natl Acad Sciences | 34 | 0.409 |
| Gustav Fischer Verlag | 33 | 0.397 |
| Hindawi Publishing Group | 31 | 0.373 |
| Korean Soc Food Science Animal Resources | 31 | 0.373 |
| Korean Society Food Science & Technology-Kosfost | 31 | 0.373 |
| Slovak Univ Agriculture Nitra | 29 | 0.349 |
| Czech Academy Agricultural Sciences | 27 | 0.325 |
| Soc Bioscience Bioengineering Japan | 26 | 0.313 |
| KLUWER ACADEMIC PUBL | 25 | 0.301 |
| Karger | 25 | 0.301 |
| Royal Soc Chemistry | 25 | 0.301 |
| Soc Brasileira Microbiologia | 25 | 0.301 |
| Natl Research Council Canada | 23 | 0.277 |
| Wageningen Academic Publishers | 23 | 0.277 |
| Faculty Food Technology Biotechnology | 22 | 0.265 |
| Mary Ann Liebert, Inc | 22 | 0.265 |
| SAGE | 22 | 0.265 |
| Canadian Science Publishing | 21 | 0.253 |
| Microbiol Res Foundation | 20 | 0.241 |
| Portland Press Ltd | 19 | 0.229 |
| Humana Press Inc | 18 | 0.217 |
| CANADIAN SCIENCE PUBLISHING, NRC RESEARCH PRESS | 16 | 0.193 |
| Edp Sciences S A | 16 | 0.193 |
| Food Nutrition Press Inc | 16 | 0.193 |
| Polskie Towarzystwo Mikrobiologow-Polish Society of Microbiologists | 16 | 0.193 |
| Research Journal Biotechnology | 16 | 0.193 |
| Univ Putra Malaysia Press | 16 | 0.193 |

**Table S3:** Top 50 bacteriocins research funding agencies

| Funding Agencies | Record Count | % of 8,305 |
| --- | --- | --- |
| United States Department of Health and Human Services | 352 | 4.238 |
| National Institutes of Health, NIH USA | 345 | 4.154 |
| National Natural Science Foundation of China, NSFC | 344 | 4.142 |
| Conselho Nacional De Desenvolvimento Cientifico E Tecnológico, CNPQ | 235 | 2.83 |
| Spanish Government | 218 | 2.625 |
| Coordenacao De Aperfeicoamento De Pessoal De Nivel Superior, CAPES | 181 | 2.179 |
| European Union, EU | 116 | 1.397 |
| Ministry Of Education Culture Sports Science and Technology Japan, MEXT | 112 | 1.349 |
| Natural Sciences and Engineering Research Council of Canada, NSERC | 109 | 1.312 |
| Japan Society for The Promotion of Science | 105 | 1.264 |
| Fundacao De Amparo A Pesquisa Do Estado De Sao Paulo, FAPESP | 95 | 1.144 |
| Science Foundation Ireland | 94 | 1.132 |
| NIH National Institute of Dental Craniofacial Research, NIDCR | 82 | 0.987 |
| NIH National Institute of Allergy Infectious Diseases NIAID | 81 | 0.975 |
| Grants in Aid for Scientific Research Kakenhi | 74 | 0.891 |
| UK Research Innovation, UKRI | 72 | 0.867 |
| Consejo Nacional De Investigaciones Cientificas Y Técnicas, CONICET | 71 | 0.855 |
| Nih National Institute of General Medical Sciences, NIGMS | 66 | 0.795 |
| National Science Foundation, NSF | 62 | 0.747 |
| Agencia Nacional de Promoción de la Investigación, el Desarrollo Tecnológico y la Innovación, ANPCYT | 60 | 0.722 |
| Council of Scientific Industrial Research, CSIR, India | 59 | 0.71 |
| University Grants Commission India | 58 | 0.698 |
| Fundacao De Amparo A Pesquisa Do Estado De Minas Gerais Fapemig | 56 | 0.674 |
| Consejo Nacional De Ciencia Y Tecnologia Conacyt | 53 | 0.638 |
| Biotechnology and Biological Sciences Research Council, BBSRC | 52 | 0.626 |
| National Research Foundation of Korea | 49 | 0.59 |
| German Research Foundation, DFG | 48 | 0.578 |
| Department of Biotechnology, Dbt, India | 47 | 0.566 |
| Fundacao Para A Ciencia E A Tecnología, FCT | 41 | 0.494 |
| National High Technology Research and Development Program of China | 39 | 0.47 |
| Research Council of Norway | 39 | 0.47 |
| Wellcome Trust | 39 | 0.47 |
| Fundamental Research Funds for The Central Universities | 35 | 0.421 |
| Junta De Andalucia | 35 | 0.421 |
| China Scholarship Council | 34 | 0.409 |
| Department Of Science Technology India | 33 | 0.397 |
| European Research Council, ERC | 33 | 0.397 |
| Vedecka Grantova Agentura Msvvas Sr A Sav Vega | 33 | 0.397 |
| Agence Nationale De La Recherche, ANR | 32 | 0.385 |
| Fundacao Carlos Chagas Filho De Amparo A Pesquisa Do Estado Do Rio De Janeiro Faperj | 30 | 0.361 |
| FWO | 30 | 0.361 |
| United States Department of Agriculture USDA | 29 | 0.349 |
| National Key Research and Development Program of China | 28 | 0.337 |
| Canada Research Chairs | 26 | 0.313 |
| China Postdoctoral Science Foundation | 24 | 0.289 |
| Natural Science Foundation of Zhejiang Province | 24 | 0.289 |
| Comunidad De Madrid | 22 | 0.265 |
| Ministry Of Education Youth Sports Czech Republic | 22 | 0.265 |
| European Commission Joint Research Centre | 21 | 0.253 |
| CGIAR | 20 | 0.241 |

**Table S4:** Bacteriocins research associated with sustainable development goals (SDGs)

| # | Sustainable Development Goals | Record Count | % of 8,303 |
| --- | --- | --- | --- |
| 1 | 03 Good Health and Well Being | 7277 | 87.455 |
| 2 | 02 Zero Hunger | 172 | 2.067 |
| 3 | 15 Life on Land | 123 | 1.478 |
| 4 | 13 Climate Action | 63 | 0.757 |
| 5 | 12 Responsible Consumption and Production | 46 | 0.553 |
| 6 | 14 Life Below Water | 36 | 0.433 |
| 7 | 06 Clean Water and Sanitation | 17 | 0.204 |
| 8 | 10 Reduced Inequality | 14 | 0.168 |
| 9 | 07 Affordable and Clean Energy | 6 | 0.072 |
| 10 | 01 No Poverty | 1 | 0.012 |
| 11 | 09 Industry Innovation And Infrastructure | 1 | 0.012 |
